# Supplementary material for: Participating in core outcome set development via Delphi surveys: qualitative interviews provide pointers to inform guidance
Source: BMJ Open. 2019 Nov 14;9(11):e032338. doi: 10.1136/bmjopen-2019-032338 (PMC6887093; doi:10.1136/bmjopen-2019-032338)

Supplementary File 1. Recruitment Advert

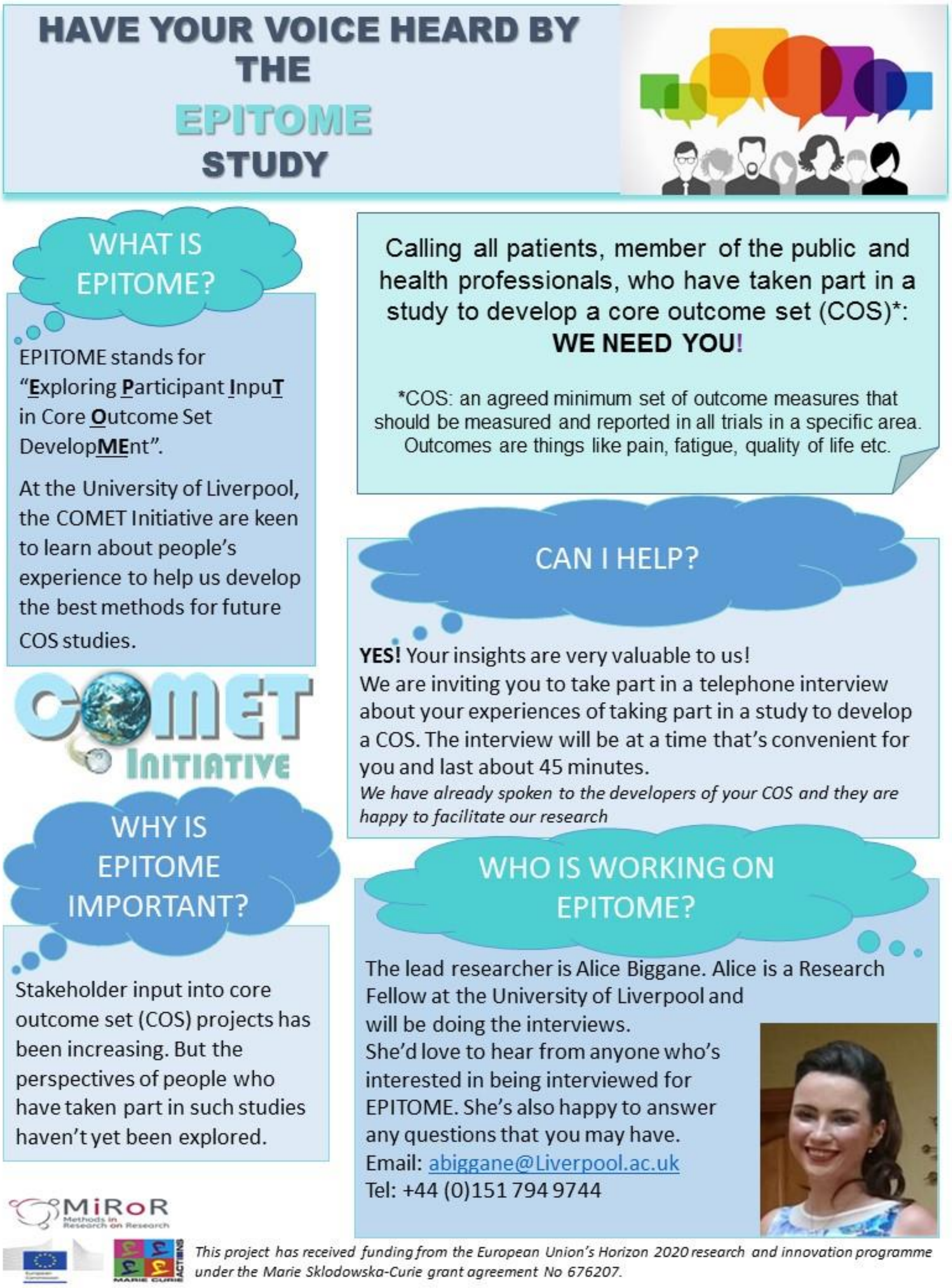

Supplement: Supplementary data [file bmjopen-2019-032338supp001.pdf]
