# Supplementary material for: Participating in core outcome set development via Delphi surveys: qualitative interviews provide pointers to inform guidance
Source: BMJ Open. 2019 Nov 14;9(11):e032338. doi: 10.1136/bmjopen-2019-032338 (PMC6887093; doi:10.1136/bmjopen-2019-032338)
Supplement: Supplementary data [file bmjopen-2019-032338supp002.pdf]

Supplementary File 2. Topic Guide

The idea of this topic guide is that the interviewer will be able to employ cognitive interviewing techniques as much as possible. By asking open and general questions it is hoped that the interviewee will retrospectively recall most of the events without interference from the interviewer, only to clarify certain aspects. However this will not always be the case and as such more detailed questions and prompts are also included. For the “engagement phase” topic of this guide, it should be noted that it may be repeated, depending on how many methods the interviewee was involved in.

Tick list:

Item	Done
Consent Form	
Expected duration of interview	
Introduction/ Explanation of process	

Topic guide (chronological)

Topic	Prompts
Background<br>*Aims: to get interviewee talking and to find out contextual information about how his/her experience of the COS development began.*	• Talk me through how you became involved in the study?<br>• How did you become aware of the study? – *Prompts: recruitment advert, methods*<br>• What were initial thoughts on it? *Prompts: Relevance, worthiness, was it explained adequately etc.*<br>• How did you make the decision to participate?<br>• Feelings about the decision
Preparation<br>*Aims: to understand how the interviewee prepared for the COS development. From their perspective and also how the study developers informed them.*	• Talk me through what happened once you decided to participate? *Prompts: what were the various stages?*<br>• What contact with the COS developers did you have before meeting them? *Prompts: post, phone calls, emails*<br>• Was this contact useful?<br>• Were you supplied with a patient information sheet? Did you look at it? *Prompts: Was it satisfactory? Did you feel like it was explained in terms you could understand?*<br>• How were outcomes described to you? *Prompts: Priorities, effect of research/ effects of treatment on life, lived experience, what is important to the patient/ what matters to them?*<br>• Did you have a clear idea about what was happening? *Prompts: Length of time, process*<br>• Was there support available to you should you need it?

Biggane AM, *et al. BMJ Open* 2019; 9:e032338. doi: 10.1136/bmjopen-2019-032338

	• Did you use the support? Was it helpful to you?
Engagement phase<br>*Aims: to elicit information regarding the process itself.*	• Talk me through what happened at the meeting/interview/ focus group/ Delphi etc.?<br>• What methods were used by the developers to elicit your thoughts and perspectives?<br>• What did you think of these methods?<br>○ Were you able to express your thoughts and feelings?<br>○ Do you feel they that your opinions were clearly re<br>• Dis you have any questions about the process? Was there support/someone to help with these? Did you access this support? Did it help?<br>○ In what capacity?<br>• For how long did your involvement in the study run? Or was it a once off?<br>○ Were you comfortable with that length of time?<br>*Prompts: too long, too short, gaps in between contact*<br>• Is there anything you would have liked to change?
Present Day<br>*Aims: to encourage the interviewee to retrospectively analyse their experience; the emotions, the process, whether the process worked or not, suggestions and messages to others.*	• Looking back on the experience what you are your thoughts about it?<br>○ Anything surprised or puzzled you?<br>○ Any suggestions for change-<br>▪ would you do it again<br>▪ would you recommend it to others?<br>• Face to face meetings with health professionals: experiences, concerns, thoughts<br>• Did you receive a copy of the final results *(if the results have been published- interviewer discretion*)?<br>• Messages to others:<br>○ Other participants, academics, developers, health professionals
Other	Anything else that wasn't covered that you think is important?
