# Supplementary material for: Participating in core outcome set development via Delphi surveys: qualitative interviews provide pointers to inform guidance
Source: BMJ Open. 2019 Nov 14;9(11):e032338. doi: 10.1136/bmjopen-2019-032338 (PMC6887093; doi:10.1136/bmjopen-2019-032338)
Supplement: Author's manuscript [file bmjopen-2019-032338.draft_revisions.pdf]

BMJ Open

BMJ Open is committed to open peer review. As part of this commitment we make the peer review history of every article we publish publicly available.

When an article is published we post the peer reviewers' comments and the authors' responses online. We also post the versions of the paper that were used during peer review. These are the versions that the peer review comments apply to.

The versions of the paper that follow are the versions that were submitted during the peer review process. They are not the versions of record or the final published versions. They should not be cited or distributed as the published version of this manuscript.

BMJ Open is an open access journal and the full, final, typeset and author-corrected version of record of the manuscript is available on our site with no access controls, subscription charges or pay-per-view fees (http://bmjopen.bmj.com).

If you have any questions on BMJ Open's open peer review process please email info.bmjopen@bmj.com

Participating in core outcome set development via Delphi surveys: Qualitative interviews provide pointers to inform guidance

Journal:	*BMJ Open*
Manuscript ID	bmjopen-2019-032338
Article Type:	Research
Date Submitted by the Author:	14-Jun-2019

Complete List of Authors:	Biggane, Alice M; University of Liverpool, Biostatistics; INSERM, U1153 Epidemiology and Biostatistics Sorbonne Paris Cité Research Center (CRESS), Methods of therapeutic evaluation of chronic diseases Team (METHODS)<br>Williamson, Paula; University of Liverpool, Biostatistics<br>Ravaud, Philippe; Universite Paris Descartes, Centre d'épidémiologie Clinique; INSERM, U1153 Epidemiology and Biostatistics Sorbonne Paris Cité Research Center (CRESS), Methods of therapeutic evaluation of chronic diseases Team (METHODS)<br>Young, Bridget; University of Liverpool, Health Services Research

Keywords:	Delphi survey, patient participation, core outcome set development

SCHOLARONE™
Manuscripts

12/06/2019
BMJ Open submission

Title page

Title:

Participating in core outcome set development via Delphi surveys: Qualitative interviews provide pointers to inform guidance

Author details:

Alice M Biggane[1,2,3]*, Paula R Williamson[1], Philippe Ravaud[2,3,4], Bridget Young[5]
[1] Department of Biostatistics, University of Liverpool, Liverpool, UK.
[2] INSERM, U1153 Epidemiology and Biostatistics Sorbonne Paris Cité Research Center (CRESS), Methods of therapeutic evaluation of chronic diseases Team (METHODS)
[3] Paris Descartes University, Sorbonne Paris Cité, Paris, France.
[4] Centre d'Epidémiologie Clinique, Hôpital Hôtel Dieu, Assistance Publique des Hôpitaux de Paris, Paris, France
[5] Department of Health Services Research, University of Liverpool, Liverpool, UK.

Abstract word count:

Manuscript Word count:

5,888

Publication type:

Research article

Key words:

Delphi survey, patient participation, core outcome set development

Funding:

This project has received funding from the European Union's Horizon 2020 research and innovation programme under the Marie Sklodowska-Curie grant agreement No 676207.

Conflict of interests:

Paula Williamson chairs the Management Group of the COMET Initiative.
Bridget Young is a member of the COMET PoPPIE (People and Public Participation, Involvement and Engagement) Working Group.
No other authors have any competing interests.

Corresponding author details:

Alice M Biggane
Department of Biostatistics,

For peer review only - http://bmjopen.bmj.com/site/about/guidelines.xhtml

12/06/2019
BMJ Open submission

University of Liverpool,
Liverpool,
UK.
abiggane@liverpool.ac.uk

Abstract:

Objectives:

To explore participants' views of Delphi surveys in core outcome set (COS) development.

Study Design and Setting

Patients and health professionals (n=24) from seven recently concluded COS studies that had involved a Delphi survey took part in semi-structured qualitative interviews (telephone and email exchange). Interviews explored participants' understanding of COS and their experiences of the Delphi survey. Analysis was thematic.

Results:

Several interviewees had previously participated in two or more COS or Delphi surveys. Those with multiple experiences of participation generally understood the purpose of COS and were satisfied with the Delphi survey. However, some interviewees who were first-time participants struggled to understand the purpose of COS and aspects of the Delphi survey, which limited their contribution and satisfaction with the study. Interviewees also differed in how they interpreted and subsequently used the written documentation provided to COS participants. Some interviewees wanted guidance regarding whose perspective to take into account when scoring outcomes and on how to use the scoring system. Interviewees reported being motivated to take part by the international and expert consensus aspects of the Delphi survey. A few interviewees reported experiencing either positive or negative emotional impacts arising from when they reviewed outcomes and stakeholder feedback.

Conclusion:

This study identifies important information that should be communicated to COS Delphi study participants. It also indicates the importance of communicating about COS Delphi studies in ways that are accessible and salient to participants, to enhance their experience of participation and make the process more meaningful for all.

Strengths and Limitations

- This is the first study to explore, in-depth, the experiences of patients and health professionals who took part in core outcome set (COS) development via the Delphi survey
- Interviewed participants had a range of experiences and perspectives

For peer review only - http://bmjopen.bmj.com/site/about/guidelines.xhtml

- The study identified ways in which participants in COS Delphi studies could be better supported
- Limitations include the retrospective nature of the interview

1. Introduction

Inconsistency in outcomes measured in clinical trials is a major concern across a multitude of health conditions, limiting the synthesis of available evidence and ability to reach reliable conclusions [1, 2].

Core outcome sets (COS) are one potential solution to this problem. A COS is a minimum set of agreed standardised outcomes which should be measured and reported in all trials in a specific condition as a minimum [3]. Three important stakeholder groups in the development of COS for trials are health professionals, patients and those who will use the COS in research, such as clinical trialists or industry[4].

Several methods are used to include stakeholders as participants in COS development, including interviews, focus groups, nominal group technique and Delphi surveys. Delphi surveys, used singularly or in combination with other methods, are the most popular method of facilitating participation [5]. These involve iterative rounds of questionnaires listing outcomes and asking participants to score the importance of each outcome. Scores are subsequently summarised across the various stakeholder groups and fed back to participants in the following round. This allows participants to consider the views of others before re-scoring each item. Furthermore, participants' views are anonymised which minimises the influence of power differentials between different stakeholders that can be problematic with direct communication between participants [6, 7]. The creation, administration and analysis of Delphi surveys is relatively inexpensive. The availability of online Delphi survey platforms allows large samples and facilitates international development of COS, thus, ensuring they are relevant globally.

However, Delphi surveys have been described as potentially intimidating for some patient participants [7] and COS developers have acknowledged a need for guidance on conducting Delphi surveys and the consensus meetings which typically follow them [8]. While recent surveys of COS participants indicate that their experiences of Delphi surveys have been generally favourable [9, 10], no research has explored in-depth the perspectives of patients and health professionals on participating in COS Delphi surveys. We therefore explored their opinions and experiences of participation to identify ways to enhance Delphi surveys for future participants in COS studies.

For peer review only - http://bmjopen.bmj.com/site/about/guidelines.xhtml

12/06/2019
BMJ Open submission

2. Methods

2.1 Research design

In the current study, EPITOME (Exploring Participant InpuT in Core Outcome Set DevelopMEnt), taking a broadly pragmatic approach we used semi-structured qualitative interviews to explore patients' and health professionals' experiences of participating in COS Delphi surveys.

2.2 Participants and recruitment

We used the responses of COS developers to a previous survey [5] to inform selection of host COS studies from which to sample interviewees. Host studies were eligible if they had involved a Delphi survey, had patient participants, included participants from more than one country, and had concluded no more than six months prior to the interview. COS developers of each host study distributed a recruitment advert (Supplementary File 1) to all stakeholders who registered for the first round of the Delphi survey. The advert invited interested individuals to contact AMB who provided a participant information sheet. For each host COS, we aimed to interview up to two patients and two health professionals. Interviewees were sent a thank you card and £15 (or currency equivalent) shopping voucher as an acknowledgement.

2.3 Data collection and analysis

Interviewees were geographically dispersed so were interviewed via telephone or email exchange. They were topic-guided and semi-structured, using a conversational approach to explore issues that we anticipated to be important, while enabling interviewees to raise areas that were important to them. Discussion with COS developers and public contributors with experience of COS development informed the development of the topic guide (Supplementary File 2), as did previous qualitative research [11]. Email exchange interviews followed a similar format asking a range of open-ended questions across topics, if necessary the interviewer, AMB followed up on responses with additional open-ended questions to further explore the interviewees' answers and comments. Furthermore, AMB, tailored questions for each interviewee by reviewing available information on the host study before the interviews.

Ongoing data analysis enabled further iterative development of the topic guide. Due to the international focus of this study, interviews were conducted via telephone or email exchange. AMB, who was a PhD student supervised by PRW and BY, conducted all interviews in English. Before starting data collection, she received training in qualitative methods. All interviewees gave informed consent. Audio-recorded interviews were transcribed verbatim, checked and anonymised before being analysed.

Analysis was initially deductive following the topic guides but became more inductive as the analysis progressed [12] and ranged from line-by-line coding, to considering whole transcripts. AMB initially read the transcripts and reflective fieldnotes that she had made immediately after each interview to inform her interpretations. A codebook was developed for the content using open coding. By grouping the codes together, recurring patterns and themes were identified and organised into categories [12]. AMB led the analysis, which she

For peer review only - http://bmjopen.bmj.com/site/about/guidelines.xhtml

periodically discussed with BY and PRW, who each read a sample of the transcripts and reviewed reports of the developing analysis. All three agreed that data saturation (the point at which new data cease to contribute to the analysis) had been reached after twenty-four interviews.

2.5 Ethics

While accepting that quality procedures cannot promise quality [13], the reporting of this study was informed by relevant guidance [14]. The study was approved by the University of Liverpool ethics committee (reference: 1969). It is important to note the authors' interests in COS development, further details of which can be found in the "Declarations" section of this manuscript, will have inevitably shaped the study and its findings.

2.6 Patient and public involvement statement

Patients and the public were involved in developing and reviewing the topic guide, recruitment advert and participant information sheets used in this study.

2.7 Definitions

We use the term 'patient' to refer to patients, carers, service users and people from organisations who represent these groups. We use 'health professional' to refer to clinicians and pharmacists. Interview excerpts shown below were selected to demonstrate the findings and our interpretations. Health professionals are indicated by "HP" and patients by "P", the COS in which they took part is indicated by "COS" and a number e.g. HP1COS1; "[.......]" indicates text removed for succinctness.

For peer review only - http://bmjopen.bmj.com/site/about/guidelines.xhtml

3. Results

3.1 COS study sampling and interviewee characteristics

We initially identified 39 potential host COS studies via the survey [5] (Supplementary File 3. Figure 1). Two further ongoing COS studies were brought to our attention by COS developers, which were not in the COMET database at the time of the survey, but were subsequently added. We contacted the developers of 20 of these COS studies in batches to inform purposive sampling to achieve maximum variation. Of these 20, we excluded 14 studies from further consideration (Figure 1). We distributed our recruitment advert, via the COS developers, to the participants in the remaining six COS studies, plus the two further studies brought to our attention, giving eight unique online COS studies. Of these we recruited participants from seven COS studies. These studies covered: geriatrics (COS1), dermatology (COS2), other (COS3), cancer (COS4), paediatrics (COS5), gynaecology and obstetrics (COS6) and otorhinolaryngology (COS7), and all aimed to recruit international participants. They varied in terms of the number of outcomes to be scored, the number of rounds, scoring system, and in the ways feedback was presented to Delphi survey participants.

For peer review only

For peer review only - http://bmjopen.bmj.com/site/about/guidelines.xhtml

Following distribution of our advert, forty participants from the seven COS studies contacted us. We did not take forward interviews with 11 of these (6 health professionals, 2 patients and 3 unknown status) as interview quotas for their COS study had been reached. Of the 29 participants invited for interview, 24 participated. Of the remaining five, two patients withdrew as they were unable to recall any details of their COS study whilst two patients and a health professional did not respond after the initial contact.

Table 1 summarises the demographic characteristics of the 24 interviewees, two interviews were completed by email exchange, and the remainder were telephone interviews. Twelve (50%) were resident in the United Kingdom (UK), four in Ireland, three in Canada, and one from each of Australia, Italy, Singapore, Spain and the Netherlands. Twenty-two interviewees described themselves as having professional occupations, two patient interviewees were retired and did not disclose their most recent occupation. Ten interviewees (three patients and seven health professionals) had previous experience of COS, Delphi surveys or both. One of the three patients with previous experience was also the patient research partner (involved in the design and conduct) of the COS development about which they were interviewed.

For peer review only

For peer review only - http://bmjopen.bmj.com/site/about/guidelines.xhtml

12/06/2019
BMJ Open submission

1

Identifier	Gender:	Age range (years):	Country:	Prior participatory experience:	
				COS development	Delphi survey
P1COS1	Male	65-74	UK	No	No
P2COS1	Female	≥75	UK	No	No
P3COS2	Female	35-44	UK	No	No
P4COS3	Female	Undisclosed	Canada	Yes	Yes
P5COS2	Male	45-54	UK	No	No
P6COS3	Female	55-64	Canada	Yes	Yes
P7COS4	Female	55-64	UK	No	No
P8COS4	Female	55-64	Netherlands	No	No
P9COS5	Female	35-44	Ireland	No	No
P10COS6	Female	45-54	Ireland	Yes[a]	Yes
P11COS7	Male	55-64	UK	No	No
P12COS7	Female	65-74	UK	No	No
P13COS2	Female	55-64	UK	No	No
HP1COS1	Female	45-54	Canada	No	Yes
HP3COS4	Male	45-54	Spain	Yes	Yes
HP4COS2	Female	35-44	Singapore	Yes	Yes
HP5COS4	Male	35-44	UK	Yes	Yes
HP6COS5	Female	55-64	UK	No[b]	No
HP7COS5	Female	25-34	Ireland	No[c]	No
HP8COS5	Female	35-44	UK	No[b]	No
HP9COS5	Female	65-74	Ireland	Yes	Yes

For peer review only - http://bmjopen.bmj.com/site/about/guidelines.xhtml

HP10COS6	Female	35-44	Italy	No	No
HP11COS6	Male	65-74	UK	Yes	Yes
HP12COS6	Female	55-64	Australia	Yes	Yes

Table 1: Interviewee demographic characteristics. [a] Interviewee was also the patient research partner of the COS study they were interviewed in relation to. [b] Two health professionals stated awareness/knowledge of COS and Delphi survey but had not participated previously. [c] One health professional was involved in an earlier phase of the COS study for which they participated in the Delphi survey.

For peer review only

For peer review only - http://bmjopen.bmj.com/site/about/guidelines.xhtml

12/06/2019
BMJ Open submission

3.2 Findings from interviews

For most interviewees, taking part in an online Delphi survey several months ago had not been a particularly salient or memorable event. Therefore, some interviewees, particularly patients, at times struggled to recall details of the host COS and so the interviewer had to provide them with brief prompts or reminders throughout the interviews. For example, P9COS5 had *"signed up to a lot of studies"* during the same time period, and asked the interviewer to remind her of what the study was about. On explaining the topic of the Delphi survey and giving some reminders of the process such as the number of rounds and the process of reviewing and scoring outcomes, P9COS5 commented that she could recall filling out only one round of the Delphi survey. Thus her interview is in relation to that only.

While all participants in each of the seven COS studies had access to resources such as information sheets (and to online videos for two of COS studies), which explained the purpose and format of the study, interviewees differed in how accurately and fully they understood the purpose of COS and the process of the Delphi survey.

3.2.1 Previous experience helped interviewees understand COS Delphi studies

As indicated in Table 1 several interviewees had previous experience of COS and Delphi surveys. In comparison to those without such experience, these interviewees generally showed a better understanding of the purpose of COS and indicated greater satisfaction with the Delphi survey. HPs with previous experience (n=7) praised COS for their importance and usefulness in research, and the Delphi survey method for its simplicity. HP5COS4 said *"that's the beauty of it, it is just not a difficult, all the hard work is done by the people that analyse the data. It is just like answering a customer service survey from Sky isn't it? Click next, next, next you just do it don't you, but I would put more effort to this than I would do a customer survey from Sky because it is more important to me."*

HPs without previous experience talked about having about read up about COS and Delphi surveys or of seeking advice from colleagues and peers to enhance their understanding of the study and prepare for their participation. For example, HP7COS5 took part in an earlier event for the same COS study at which the developers had been present; *"it made me think more fully about the bigger picture of research going forward and how these processes like the Delphi survey feed into that"* and that otherwise she *"would have approached it in a less informed way."*

Three patient interviewees also spoke about the impact of their previous experiences in COS Delphi studies. Over the course of these studies, they described their experience evolving from one of confusion during their first study to one of enjoying the process and better understanding the purpose of COS with each subsequent study *"once you get the hang of it, I really enjoy doing them because I like where it takes you"* (P6COS3). P10COS6 spoke of not having a *"bull's notion what is going on"* in earlier studies with regard to both the purpose and method of COS development and had *"to do a lot of online research myself to learn",* despite receiving information sheets for each study. Reflecting on this evolving experience of COS and Delphi surveys during her interview, she suggested that providing participants with

For peer review only - http://bmjopen.bmj.com/site/about/guidelines.xhtml

a visual synopsis of the purpose of COS and Delphi survey method from the outset of a study would be helpful: *"I would have assimilated the message much quicker."*

Patient interviewees (n=9) with no previous experience, varied in their understanding of the purpose of the Delphi survey. The comments of some showed that they understood the Delphi survey's purpose was to reach consensus on which core outcomes to include. For example, P7COS4 explained the study was: *"looking at how people felt with their recovery […] what they went through and what they were left with and how important those were to the person involved*." In contrast, others such as P8COS4 described the Delphi survey's purpose more vaguely as to gather a *"broad base of information on how many different people experience the treatment."* Moreover, she did not talk about the process in terms of prioritising the outcomes listed or reaching consensus amongst stakeholders. P1COS1 was confused about whether his study was complete or if he should expect further rounds of the survey: *"I don't even know that you could say a line had been drawn under it*." P11COS7 reflected on whether he *"could have done more to understand how the process worked earlier on. Particularly with the […] expert involvement, I now understand so next time I shall be even better at it"* and suggested *"a practice run"* would have been useful before entering the actual study. In a few cases participants indicated that their lack of understanding had influenced their overall experience of participation, *"I think one of my real concerns is that I didn't really contribute anything to the research because I really wasn't sure what I was doing"* (P2COS1).

3.2.2 Helping participants understand the purpose and process of Delphi surveys - one size does not fit all

The findings indicate that interviewees had different needs for support to aid their understanding of the purpose and process of COS Delphi surveys. P3CSO2 and P5COS2 were two first-time patient participants. They both received the same study documentation and said they reviewed it. However, their accounts indicated that they differed in their understanding of the documentation, and these differences influenced their contributions to and experiences of the study.

P5COS2 thought the study documentation he received was *"appropriate"*, elaborating *"I have worked in the past in IT, in pharmaceuticals, in politics[…]so I am quite happy to see text that is fairly technical in nature or fairly clinical in nature and you know that is something I find easy enough to get to grips with."* He thought that the study "*was a very constructive thing to do. And I could see personally, something like that being done prior to any clinical trial, so that the end points of the clinical trial […] look at, you know how beneficial say a product is from the patient's perspective*."

In contrast, P3COS2 who worked in marketing commented that she *"didn't understand the terminology"* in the documents and as a result described being *"switched off from the process element […] psychologically I was just focussed on taking part and having my say."* She wondered if the study and its data would get *"stored away somewhere in a filing cabinet and forgotten about [….] I think what was lacking in the communication is how this is going to actually practically inform future research. And maybe that is my lack of understanding of how these sort of surveys work, and how these outcome surveys work, I don't really get, how*

For peer review only - http://bmjopen.bmj.com/site/about/guidelines.xhtml

that will translate into future treatments." In response to P3COS2's comment, the interviewer explained that COS were used as minimum sets of outcomes in clinical trials so that evidence can be compared across studies and inform decision making regarding treatments. The interviewer added that the Delphi survey was a method to develop the COS by seeking consensus amongst relevant experts including patients. In response, P3 recalled that she had received information to that effect in the study documentation before adding *"I really wish that had been captured in the communication a bit more clearly […] maybe I'd have done things differently*."

For peer review only

For peer review only - http://bmjopen.bmj.com/site/about/guidelines.xhtml

3.2.3 Representation in the Delphi survey- who and when

Both HP and patient interviewees raised the issue of "who they should be representing?" when completing the Delphi survey. They questioned whether they should try to think or imagine what outcomes fellow patients or HPs would likely prioritise when scoring the outcomes study, or whether they should focus only on their own opinions and priorities. None reported receiving guidance on this.

P5COS2 thought *"it can only be a genuine result if everybody says what they personally feel"* and "*trying to guess* [how others feel]*"* would defeat the objective. This contrasts with P7COS4, a female who described trying to answer the outcomes section of that was applicable to males only: "*I just thought well if I was in that situation I will answer it as if I was that person maybe you know. […] Yes maybe I shouldn't have done that."*

In COS3, both patient interviewees were also advocates in a relevant patient organisation, and both had previous experience of COS Delphi studies. P6 described how she *"learned very early on"* to answer from her own perspective. Conversely, P4 drew on her knowledge of the perspectives of other patients from discussions she had had through her work with the patient organisation *"I do try to work in their concerns and the issues that they have."* She added that COS developers should consider how the different phases in a patient's journey and their life could affect the way they scored outcomes: *"my priorities are different now, than they were when I was diagnosed over 30 years ago […] you know different things would have affected me. […] over the years with the chronic disease you learn to live with it and adapt to it, so […] yes I think that can affect your responses too."*

HPs touched on similar issues regarding who to represent when scoring outcomes, although compared to patients, this was less prominent in their accounts. HP1COS1, was an academic, a service provider and a policy maker. Referring to both her experiences as a professional and her personal opinions, she explained that she drew on *"a bit of both"* when scoring outcomes. Similarly, HP11COS6, an academic and service provider, explained *"it was a mixture of, of relating it to myself and relating it to patients. But I was, even when I was relating it to myself I was relating it to me thinking of myself as a patient or the father of a patient or something like that."*

For peer review only - http://bmjopen.bmj.com/site/about/guidelines.xhtml

3.2.4 Motivational and emotional aspects of participation

A few patients and HPs talked about the motivational and emotional aspects of their participation.

Health professionals praised the Delphi survey method of COS development for its consensual and collaborative approach, and cited the opportunity to learn from international colleagues as one of the motivations for participating. They also spoke of their belief in the importance of COS in their field and their desire to contribute.

Patients described taking part out of gratitude for a study in which they could contribute their lived experience. P8COS4 talked about how her illness was *"rare"* and how information and research on the illness was limited *"so it was great for us* (other patients) *and for me specifically you know to fill in something that was specifically to do with my* (illness)", she further elaborated that the COS study *"made us feel someone was listening or someone was going to help us."* P3COS2 talked about how she felt *"happy"* to be included in research relevant to her, as she was outside the age range that was typical for patients with the health condition concerned. Similarly, P5COS2 *"thought it was quite exciting the fact that they would ask regular kind of sufferers of particular problems what do you think should be included in a trial. What outcomes do you think are important and everything and getting feedback from people outside the scientific community. I thought was quite cool and as somebody who suffers from various medical conditions the ability for me to give my input on what I think is important to a patient."*

P6CO3 had participated in multiple COS Delphi studies. She described her enthusiasm for the Delphi survey as a motivation to participate: *"every time I do them, I enjoy them more I really, really like the process"* and her willingness to participate in studies that used the resulting COS: *"you might have a preconceived notion of what something should be, or perspective on what something should be, or what the final product should look like, and it takes you in a different direction and if you just kind of you know let go and let it take you where it takes you through the questions and the feedback and everything I think it is a really interesting way of coming up with a list and I think it is a really true list."*

Two patients and one health professional indicated that reviewing the list of outcomes had affected them emotionally. Speaking of when she reviewed the scores provided by fellow participants in the second round of P8COS4 commented that she had: *"changed some of my answers on the second round, when I was thinking about having a possible* (intervention removed) *then I was like oh, I wouldn't want that at all […] I was sort of realising that I was grateful for where I was basically."* HP7COS5 said that when reviewing the fellow participants' feedback *"there were definitely moments of almost insecurity I suppose because you are aware, […] you are in amongst a group of other people who are very familiar with this field and experts […]"* She described initially feeling uncertain about her answers: *"it is ok to obviously be encouraged to check back on yourself and to be really thoughtful when you are kind of giving those sorts of answers [….] so I think there was a little bit of both an awareness of needing to stay objective but there was certainly a more subjective, emotive aspect to seeing how other people were answering."*

For peer review only - http://bmjopen.bmj.com/site/about/guidelines.xhtml

P2COS1 spoke of how reviewing the outcomes as part of the COS study had made her aware of outcomes that she had not previously realised were associated with her condition and treatment: *"A lot of the outcomes I would never have thought of those as outcomes from the sort of medication I am on if you see what I mean."* She described how this had affected her: *"I am seriously worried about that. […] I was given no indication* [by healthcare provider] *[…] that I need to be careful."*

For peer review only

For peer review only - http://bmjopen.bmj.com/site/about/guidelines.xhtml

3.2.5 Scoring system

The scoring systems in the seven host COS studies used either a 9- (n=6) or 5- (n=1) point Likert scale. In five of the COS that used a 9-point Likert, scores were further differentiated as: 1-3 'Not important' (n=4) or 'Limited importance' (n=1), 4-6 'Important but not critical', 7-9 'Critical'. In the sixth, the anchor descriptions were 'not at all important' (1) and 'extremely important' (9). In the COS that used a 5-point Likert scale, participants were asked to rate their level of agreement on a series of statements regarding potential outcomes, with scores labelled: 1 Strongly disagree, 2 Disagree, 3 Ambivalent, 4 Agree, 5 Strongly agree.

Several interviewees did not comment on the scoring system during their interview. Those who did comment varied from praising or indicating satisfaction with the scoring system, to wanting a system with fewer categories and further guidance on how to apply the scale, although the majority of interviewees were positive about the scales used in COS Delphi studies that they had taken part in. Those who expressed satisfaction with the 9-point scales, indicated that they were familiar with using these: *"I am usually happy with Likert scales so, fine"* (HP12COS6), while another interviewee summed up her experience of the scales as *"not a big deal"* (P4COS3).

Interviewees who took part in a COS that used a 9-point scale and liked it praised the wide range of options and the three distinct bands as helpful. For example HP9COS5 commented *"I liked the way they set it out in that they were, you know while it was 9 it was important, not so important and least important so that even within those categories one could actually subdivide them, and I actually think I liked that. Sometimes you know you are asked you know, should something be important, and there are kind of gradations within importance, and so I think that for me I liked that subdivision. It gave me a little bit more flexibility."* P7COS4 noted *"grading it you know, systematically up from 1 to 9 so yes that was useful because it give you, although a lot of my scores were up on the higher range there were a couple of lower ones so I think the having 1 to 9 was a good idea."*

Other interviewees had a preference for fewer categories. Speaking of the 9-point scale in her study, P2COS1 commented "*I really don't think a score from 1 to 10 is realistic. […] maybe if you are a very skilled researcher yourself you might be able to deal in that level of gradation but I don't think the vast majority of us can. I think, you know, a 5 point rating scale is the most that most of us could do. You know with any degree of accuracy."* Similarly, also speaking of the 9 point scale HP8COS5 said "*what is the difference between a 6 and 7, you know what I mean if it is just sort of all in the middle of the road […] so whether or not it could have been less numbers to help make a more definitive answer."* However, like other interviewees who had a preference for a scale with fewer categories she acknowledged *"there might be reasonings behind why you have got 0-9 and that type of thing*." While some interviewees found the three bands on the 9-point scale helpful, responses from some health professionals and patients indicated that further guidance and support is needed to help them use the 9-point scale. Similarly P11COS7, a first time patient participant, raised the difficulties he experienced in *"connecting physical sensations with a numerical value"* when relating his physical symptoms to scoring outcomes. He added that this *"produces a certain anxiety between whether you pick 5, 6 or 7."*

For peer review only - http://bmjopen.bmj.com/site/about/guidelines.xhtml

HP6COS5 was the only interviewee who compared the scoring system to other methods of prioritisation when she flagged her overall preference for a numerical scale when scoring a long list of items in comparison to ranking them *"if I had been given the list and said you know rate these 1 to 20 it would have been harder to do."*

For peer review only - http://bmjopen.bmj.com/site/about/guidelines.xhtml

12/06/2019
BMJ Open submission

4. Discussion

4.1 Summary of findings

We found that while some interviewees understood the purpose of COS and the Delphi survey, others struggled to understand the purpose and aspects of the Delphi survey method which in turn influenced their contribution and experience of the study. The accounts of the interviewees indicate that COS participants would benefit from further guidance and support.

Interviewees could be broadly separated into two categories; those with and without previous experience of COS development and/or Delphi surveys. The accounts of those with previous experience, both health professionals and patients, showed they had a good understanding of the purpose of COS and were satisfied with the Delphi survey as a method of participation. Health professionals without previous experience reported engaging with relevant literature and colleagues prior to and during participation, thus enhancing their understanding and experience. In contrast, the accounts of patients without previous experience indicated considerable variation with some showing good understanding, while others understood little of the study and its purpose. Aspects that the latter group struggled with included understanding that the Delphi survey aimed to achieve consensus amongst stakeholders, applying the scoring system and knowing whose views to represent when participating. This limited their engagement and interpretation of the documentation they had received from COS developers, and their input and experience of COS development.

The importance of representing of all relevant stakeholder groups including patients in COS development [4, 7] is increasingly recognised, as it is in wider health research [16-18]. There is also growing appreciation of the importance of supporting their participation in ways that are meaningful, thus avoiding tokenism and enhancing the credibility and validity of the resulting research [19, 20]. However, our findings suggest that not all the interviewees thought their participation in COS development was meaningful, as the purpose and process of the study was communicated in ways that were not accessible for them. Theory surrounding health literacy describes its role in patient empowerment and advocates for information to be made accessible to all patients in appropriate formats [21-24] This is particularly important for patient participants in COS development, most of whom will not have taken part in this type of research previously nor have access to the literature or colleagues to illuminate the process. A few patient interviewees in this study indicated that they saw understanding COS Delphi studies as their personal responsibility or felt uncomfortable with their limited of understanding. However, when asking patients to participate in COS studies developers are inviting them to the world of research [7], thus, it is the responsibility of the COS development community to ensure the guidance and support is in place to allow meaningful participation. There has been a rapid expansion in the number of COS being developed, with an associated rapid increase in the number including patients in Delphi surveys. Our findings indicate that this expansion has perhaps outpaced the development of relevant guidance for Delphi studies to enable meaningful participation for all.

For peer review only - http://bmjopen.bmj.com/site/about/guidelines.xhtml

12/06/2019
BMJ Open submission

This study points to specific areas where further guidance and support is required to communicate the purpose of COS and the process of the Delphi survey. This complements the findings of two recent surveys of COS Delphi study participants which indicated that they benefit from repeated guidance on principles of core outcome set development during the rounds, that reminders about these principles were acceptable [10], and that recruitment and retention of participants is more likely with personalised communication [9]. To date the most common way of providing participant information regarding a research project is via written documentation. Much research has indicated poor health literacy is prevalent [25-28], thus the importance of ensuring plain language communication cannot be underestimated. However, this study's findings suggest not only is plain language communication required, but also further consideration of how to explain the purpose of COS in ways that are relatable and salient to patients. This explanation and delivery could make use of visual, written and auditory methods, such as analogies, infographics, visual metaphors, digital stories and other narrative forms. The most appropriate method or combination of methods is likely to depend on the population and health condition to which the COS will be relevant. The use of visual resources have been documented in other healthcare areas such as health promotion [29], patient education [30] and nursing training [31]. In COS development demonstration videos of the Delphi survey enhanced participant retention to the study [9]. The COMET website provides resources to help developers facilitate participation, including documents explaining COS in plain English and an animation video (http://www.comet-initiative.org/resources/PlainLanguageSummary), co-produced with members of the public.

This study also indicates areas in which further research and direction would be useful. The issues raised by interviewees regarding how to apply the scoring system, point to the need for better communication. The 9-point Likert scoring system where items are graded in accordance to their level of importance is a common method, recommended by the Grading of Recommendations Assessment, Development and Evaluation (GRADE) Working Group [32]. There are statistical considerations in support of using a longer scale including the ability to calculate variance in scores. Thus, it is important that participants in COS Delphi studies have the information and support they need to apply this system. Involving patients and members of the public as active research partners would provide a patient perspective on the suitability of different aspects of the COS study from design to conclusion, including helping with the development of appropriate documentation, resources and support [7, 9].

Interviewees also raised the issue of whose perspective to take into account when scoring outcomes. Pending further research, we would recommend that in the first round of the Delphi survey COS developers ask participants to score according to their own individual perspective, not score according to the perspective of others. In the second or subsequent rounds participants should be asked to reflect on the scores of other participants, while being clear that they do not have to change their own scores. Having reflected participants should be asked to score according to their current view of what a COS in that specified health condition should include [33]. Participants can be encouraged to score outcomes they have no experience of to date, but may experience in the future, although an "unable to score"

For peer review only - http://bmjopen.bmj.com/site/about/guidelines.xhtml

option or equivalent should also be provided for each outcome. A key exception to participants scoring from their own individual perspective is when carers act as proxy respondents in COS studies. In health research on certain patient populations there is often no alternative to using proxies [34, 35], yet there is evidence of discrepancies in how proxies prioritise outcomes compared to patients themselves [36, 37]. During the first round of COS Delphi studies proxies should score according to what they anticipate is the perspective of the patient and not from their own perspective as a carer, and follow the same advice as other participants in subsequent rounds. Thus, COS developers should consider which proxies can provide a valid opinion on the anticipated perspective of the patient and how best to support this type of participation.

Some interviewees described the motivation and emotions associated with their participation. Understanding that participants are motivated to engage in COS development out of desire to contribute to the research topic and satisfaction with the Delphi survey's collaborative and international approach will be useful to COS developers when advertising and recruiting participants to their study. The emotional impact of participation requires consideration from developers and researchers when designing and conducting their COS studies to optimise the experience of participants and minimise any negative impacts on them.

4.2. Strengths and weaknesses of the study

This study has provided insights into COS development via Delphi surveys from the perspective of participants. As previously noted, participation in the COS Delphi studies was not a particularly salient event for interviewees, however during their interviews they were provided with tailored prompts and reminders as needed.

This study only describes the experiences of participants who agreed to be interviewed, recruited from seven COS studies and limited to English-speakers. Thus, while saturation was reached within our sample, it is possible that not all participant experiences are represented here. However, by purposively sampling across a range of COS studies, we anticipate that our findings will be broadly transferable to other COS studies. Moreover, our interviewees were international, reflecting the increasing international development of COS.

5. Conclusion

This study's findings contribute to the growing evidence base on participation in COS development. The identification of areas where participants need enhanced guidance and support will be useful to future COS developers when planning their studies, enabling them to recruit and support participants towards a meaningful and positive experience of COS Delphi studies.

For peer review only - http://bmjopen.bmj.com/site/about/guidelines.xhtml

12/06/2019
BMJ Open submission

Declarations

Ethics approval and consent to participate

Ethical approval was granted from Health and Life Sciences Committee on Research Ethics (Human participants, tissues and databases) at The University of Liverpool, on 22/06/2017 (reference 1969). All interviewees were provided with full written information prior to interview commencement. All interviewees gave audio-recorded or written informed consent prior to commencing the interview and were free to end the interview at any time without providing a reason.

Consent for publication

Not applicable

Availability of data and materials

Not applicable

Competing interests

PRW helped to found the Core Outcome Measures in Effectiveness Trials (COMET) Initiative in 2010, which promotes the development and application of COS. COMET developed software, DelphiManager, for conducting online Delphi surveys.

BY has interests in the development of COS, since 2015 she has co-chaired the COMET People and Patient Participation Involvement and Engagement (PoPPIE) Working Group.

No other authors have any competing interests.

Funding

This project is a part of a MiRoR (Methods in Research on Research) funded PhD, which is being undertaken by AB. MiRoR has received funding from the European Union's Horizon 2020 research and innovation programme under the Marie Sklodowska-Curie grant agreement No 676207.

Authors' contributions

All authors have read and approved the final version of this manuscript. AB was the lead researcher on this project and was responsible for the preparation and drafting of the protocol, data collection and analysis and writing of this manuscript. PRW is a co-investigator and contributed to project conception, design, protocol writing, analysis, writing and proofreading of this manuscript. PR is a co-investigator and contributed to the methodology used in creating the sampling framework. BY is principal investigator on this project and is responsible for its design, protocol writing, analysis, writing and proofreading of this manuscript.

For peer review only - http://bmjopen.bmj.com/site/about/guidelines.xhtml

Acknowledgements

The authors would like to thank the COS developers and the interviewees for their time and input. The authors would like to acknowledge the Methods in Research on Research (MiRoR) network for their support and guidance, in particular Van Thu Nguyen, Ketevan Glonti and Melissa Sharp. They authors are also grateful to Camila Olarte Parra and Els Goetghebeur for their thoughtful comments and suggestions on the final draft of the manuscript.

For peer review only

For peer review only - http://bmjopen.bmj.com/site/about/guidelines.xhtml

12/06/2019
BMJ Open submission

References

[1] Chalmers I, Glasziou P. Avoidable waste in the production and reporting of research evidence. Lancet. 2009;374.
[2] Chong LS, Sautenet B, Tong A, Hanson CS, Samuel S, Zappitelli M, et al. Range and Heterogeneity of Outcomes in Randomized Trials of Pediatric Chronic Kidney Disease. The Journal of Pediatrics. 2017;186:110-7. e11.
[3] Williamson PR, Altman DG, Blazeby JM, Clarke M, Devane D, Gargon E, et al. Developing core outcome sets for clinical trials: issues to consider. Trials. 2012;13:132.
[4] Kirkham JJ, Davis K, Altman DG, Blazeby JM, Clarke M, Tunis S, et al. Core Outcome Set-STAndards for Development: The COS-STAD recommendations. PLoS Medicine. 2017;14:e1002447.
[5] Biggane AM, Brading L, Ravaud P, Young B, Williamson PR. Survey indicated that core outcome set development is increasingly including patients, being conducted internationally and using Delphi surveys. Trials. 2018;19:113.
[6] Sinha IP, Smyth RL, Williamson PR. Using the Delphi technique to determine which outcomes to measure in clinical trials: recommendations for the future based on a systematic review of existing studies. PLoS Medicine. 2011;8:e1000393.
[7] Young B, Bagley H. Including patients in core outcome set development: issues to consider based on three workshops with around 100 international delegates. Research Involvement and Engagement. 2016;2:25.
[8] Gargon E, Williamson PR, Young B. Improving core outcome set development: qualitative interviews with developers provided pointers to inform guidance. Journal of Clinical Epidemiology. 2017;86:140-52.
[9] Hall DA, Smith H, Heffernan E, Fackrell K. Recruiting and retaining participants in e-Delphi surveys for core outcome set development: Evaluating the COMiT'ID study. PloS ONE. 2018;13:e0201378.
[10] Turnbull AE, Dinglas VD, Friedman LA, Chessare CM, Sepúlveda KA, Bingham III CO, et al. A survey of Delphi panelists after core outcome set development revealed positive feedback and methods to facilitate panel member participation. Journal of Clinical Epidemiology. 2018;102:99-106.
[11] Ritchie J, Lewis J, Nicholls CM, Ormston R. Qualitative research practice: A guide for social science students and researchers: Sage; 2013.
[12] Braun V, Clarke V, Terry G. Thematic analysis. Qual Res Clin Health Psychol. 2014;24:95-114.
[13] Barbour RS. Checklists for improving rigour in qualitative research: a case of the tail wagging the dog? BMJ: British Medical Journal. 2001;322:1115.
[14] O'Brien BC, Harris IB, Beckman TJ, Reed DA, Cook DA. Standards for reporting qualitative research: a synthesis of recommendations. Academic medicine : journal of the Association of American Medical Colleges. 2014;89:1245-51.
[15] Voigt P, Von dem Bussche A. The EU General Data Protection Regulation (GDPR). A Practical Guide, 1st Ed, Cham: Springer International Publishing. 2017.
[16] Boote J, Baird W, Beecroft C. Public involvement at the design stage of primary health research: a narrative review of case examples. Health policy (Amsterdam, Netherlands). 2010;95:10-23.
[17] Ocloo J, Matthews R. From tokenism to empowerment: progressing patient and public involvement in healthcare improvement. BMJ Qual Saf. 2016;25:626-32.
[18] Supple D, Roberts A, Hudson V, Masefield S, Fitch N, Rahmen M, et al. From tokenism to meaningful engagement: best practices in patient involvement in an EU project. Research Involvement and Engagement. 2015;1:5.
[19] Smith J, Dransfield A. Patient and carer involvement in healthcare education, service delivery and research: avoiding tokenism. Royal College of Nursing; 2019.
[20] Rolfe DE, Ramsden VR, Banner D, Graham ID. Using qualitative Health Research methods to improve patient and public involvement and engagement in research. Research Involvement and Engagement. 2018;4:49.

For peer review only - http://bmjopen.bmj.com/site/about/guidelines.xhtml

12/06/2019
BMJ Open submission

[21] Stableford S, Mettger W. Plain language: a strategic response to the health literacy challenge. Journal of public health policy. 2007;28:71-93.
[22] Sørensen K, Van den Broucke S, Fullam J, Doyle G, Pelikan J, Slonska Z, et al. Health literacy and public health: A systematic review and integration of definitions and models. BMC Public Health. 2012;12:80.
[23] Smith SK, Dixon A, Trevena L, Nutbeam D, McCaffery KJ. Exploring patient involvement in healthcare decision making across different education and functional health literacy groups. Social science & medicine. 2009;69:1805-12.
[24] Nutbeam D. The evolving concept of health literacy. Social science & medicine. 2008;67:2072-8.
[25] Sørensen K, Pelikan JM, Röthlin F, Ganahl K, Slonska Z, Doyle G, et al. Health literacy in Europe: comparative results of the European health literacy survey (HLS-EU). European Journal of Public Health. 2015;25:1053-8.
[26] Nutbeam D. Health literacy as a public health goal: a challenge for contemporary health education and communication strategies into the 21st century. Health Promotion International. 2000;15:259-67.
[27] Batterham R, Hawkins M, Collins P, Buchbinder R, Osborne R. Health literacy: applying current concepts to improve health services and reduce health inequalities. Public Health. 2016;132:3-12.
[28] Paasche-Orlow MK, Parker RM, Gazmararian JA, Nielsen-Bohlman LT, Rudd RR. The prevalence of limited health literacy. Journal of general internal medicine. 2005;20:175-84.
[29] Briant KJ, Halter A, Marchello N, Escareno M, Thompson B. The Power of Digital Storytelling as a Culturally Relevant Health Promotion Tool. Health promotion practice. 2016;17:793-801.
[30] Skelton SL, Waterman AD, Davis LA, Peipert JD, Fish AF. Applying best practices to designing patient education for patients with end-stage renal disease pursuing kidney transplant. Progress in Transplantation. 2015;25:77-90.
[31] Christiansen A. Storytelling and professional learning: A phenomenographic study of students' experience of patient digital stories in nurse education. Nurse Education Today. 2011;31:289-93.
[32] Guyatt GH, Oxman AD, Kunz R, Vist GE, Falck-Ytter Y, Schunemann HJ. What is "quality of evidence" and why is it important to clinicians? Bmj. 2008;336:995-8.
[33] Linstone HA, Turoff M. The delphi method: Addison-Wesley Reading, MA; 1975.
[34] Sprangers MA, Aaronson NK. The role of health care providers and significant others in evaluating the quality of life of patients with chronic disease: a review. Journal of clinical epidemiology. 1992;45:743-60.
[35] Sinha IP, Altman DG, Beresford MW, Boers M, Clarke M, Craig J, et al. Standard 5: selection, measurement, and reporting of outcomes in clinical trials in children. Pediatrics. 2012;129:S146-S52.
[36] Dinglas VD, Gifford JM, Husain N, Colantuoni E, Needham DM. Quality of life before intensive care using EQ-5D: patient versus proxy responses. Critical care medicine. 2013;41:9-14.
[37] Arons AM, Krabbe PF, Schölzel-Dorenbos CJ, van der Wilt GJ, Rikkert MGO. Quality of life in dementia: a study on proxy bias. BMC Medical Research Methodology. 2013;13:110.

For peer review only - http://bmjopen.bmj.com/site/about/guidelines.xhtml

Supplementary File 1. Recruitment Advert

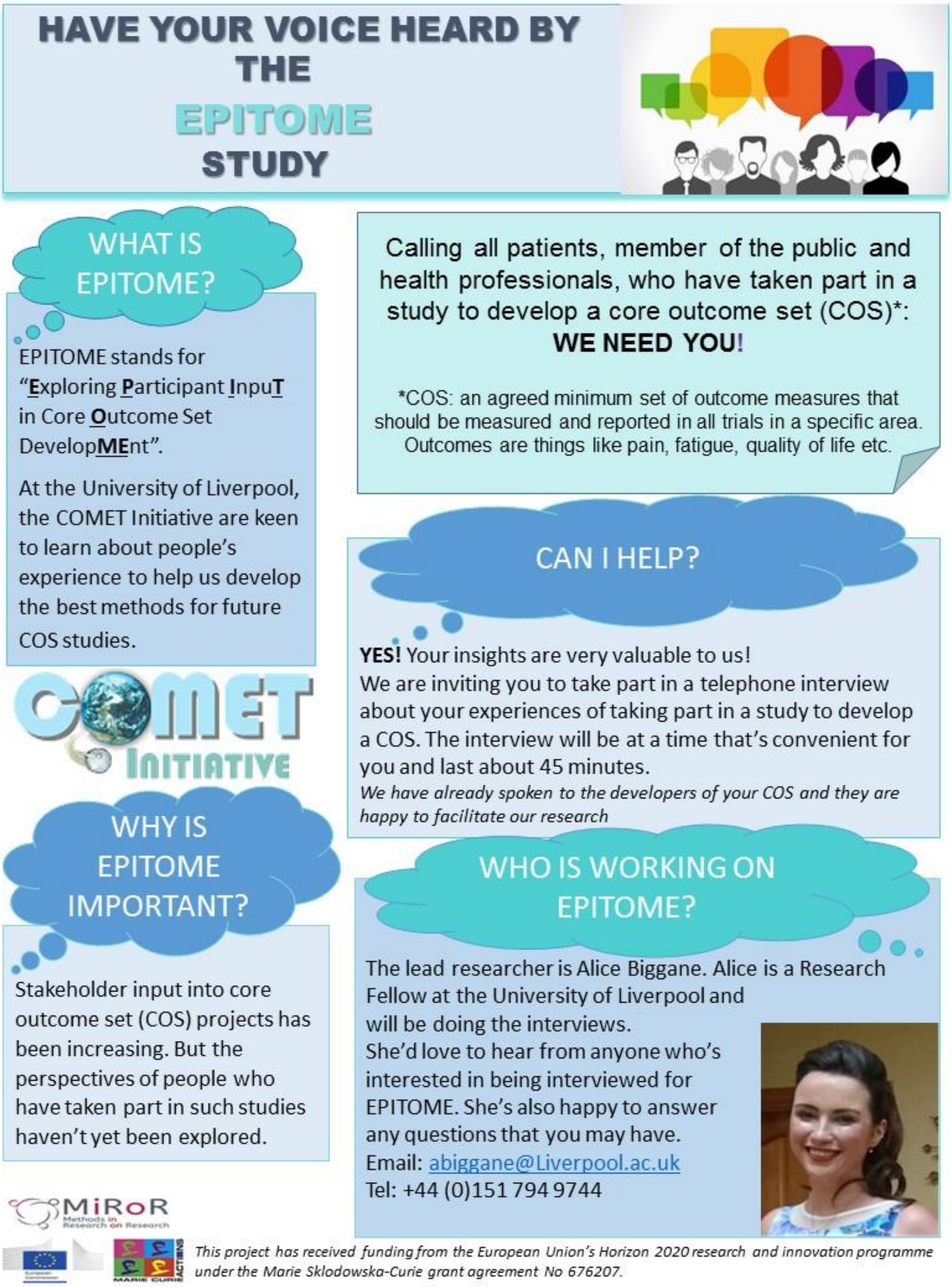

Supplementary File 2. Topic Guide

The idea of this topic guide is that the interviewer will be able to employ cognitive interviewing techniques as much as possible. By asking open and general questions it is hoped that the interviewee will retrospectively recall most of the events without interference from the interviewer, only to clarify certain aspects. However this will not always be the case and as such more detailed questions and prompts are also included. For the “engagement phase” topic of this guide, it should be noted that it may be repeated, depending on how many methods the interviewee was involved in.

Tick list:

Item	Done
Consent Form	
Expected duration of interview	
Introduction/ Explanation of process	

Topic guide (chronological)

Topic	Prompts
Background<br>*Aims: to get interviewee talking and to find out contextual information about how his/her experience of the COS development began.*	• Talk me through how you became involved in the study?<br>• How did you become aware of the study? – *Prompts: recruitment advert, methods*<br>• What were initial thoughts on it? *Prompts: Relevance, worthiness, was it explained adequately etc.*<br>• How did you make the decision to participate?<br>• Feelings about the decision
Preparation<br>*Aims: to understand how the interviewee prepared for the COS development. From their perspective and also how the study developers informed them.*	• Talk me through what happened once you decided to participate? *Prompts: what were the various stages?*<br>• What contact with the COS developers did you have before meeting them? *Prompts: post, phone calls, emails*<br>• Was this contact useful?<br>• Were you supplied with a patient information sheet? Did you look at it? *Prompts: Was it satisfactory? Did you feel like it was explained in terms you could understand?*<br>• How were outcomes described to you? *Prompts: Priorities, effect of research/ effects of treatment on life, lived experience, what is important to the patient/ what matters to them?*<br>• Did you have a clear idea about what was happening? *Prompts: Length of time, process*<br>• Was there support available to you should you need it?

For peer review only - http://bmjopen.bmj.com/site/about/guidelines.xhtml

	• Did you use the support? Was it helpful to you?
Engagement phase<br>*Aims: to elicit information regarding the process itself.*	• Talk me through what happened at the meeting/interview/ focus group/ Delphi etc.?<br>• What methods were used by the developers to elicit your thoughts and perspectives?<br>• What did you think of these methods?<br>○ Were you able to express your thoughts and feelings?<br>○ Do you feel they that your opinions were clearly re<br>• Dis you have any questions about the process? Was there support/someone to help with these? Did you access this support? Did it help?<br>○ In what capacity?<br>• For how long did your involvement in the study run? Or was it a once off?<br>○ Were you comfortable with that length of time?<br>*Prompts: too long, too short, gaps in between contact*<br>• Is there anything you would have liked to change?
Present Day<br>*Aims: to encourage the interviewee to retrospectively analyse their experience; the emotions, the process, whether the process worked or not, suggestions and messages to others.*	• Looking back on the experience what you are your thoughts about it?<br>○ Anything surprised or puzzled you?<br>○ Any suggestions for change-<br>▪ would you do it again<br>▪ would you recommend it to others?<br>• Face to face meetings with health professionals: experiences, concerns, thoughts<br>• Did you receive a copy of the final results *(if the results have been published- interviewer discretion)*?<br>• Messages to others:<br>○ Other participants, academics, developers, health professionals
Other	Anything else that wasn't covered that you think is important?

For peer review only

For peer review only - http://bmjopen.bmj.com/site/about/guidelines.xhtml

Supplementary File 3. Figure 1

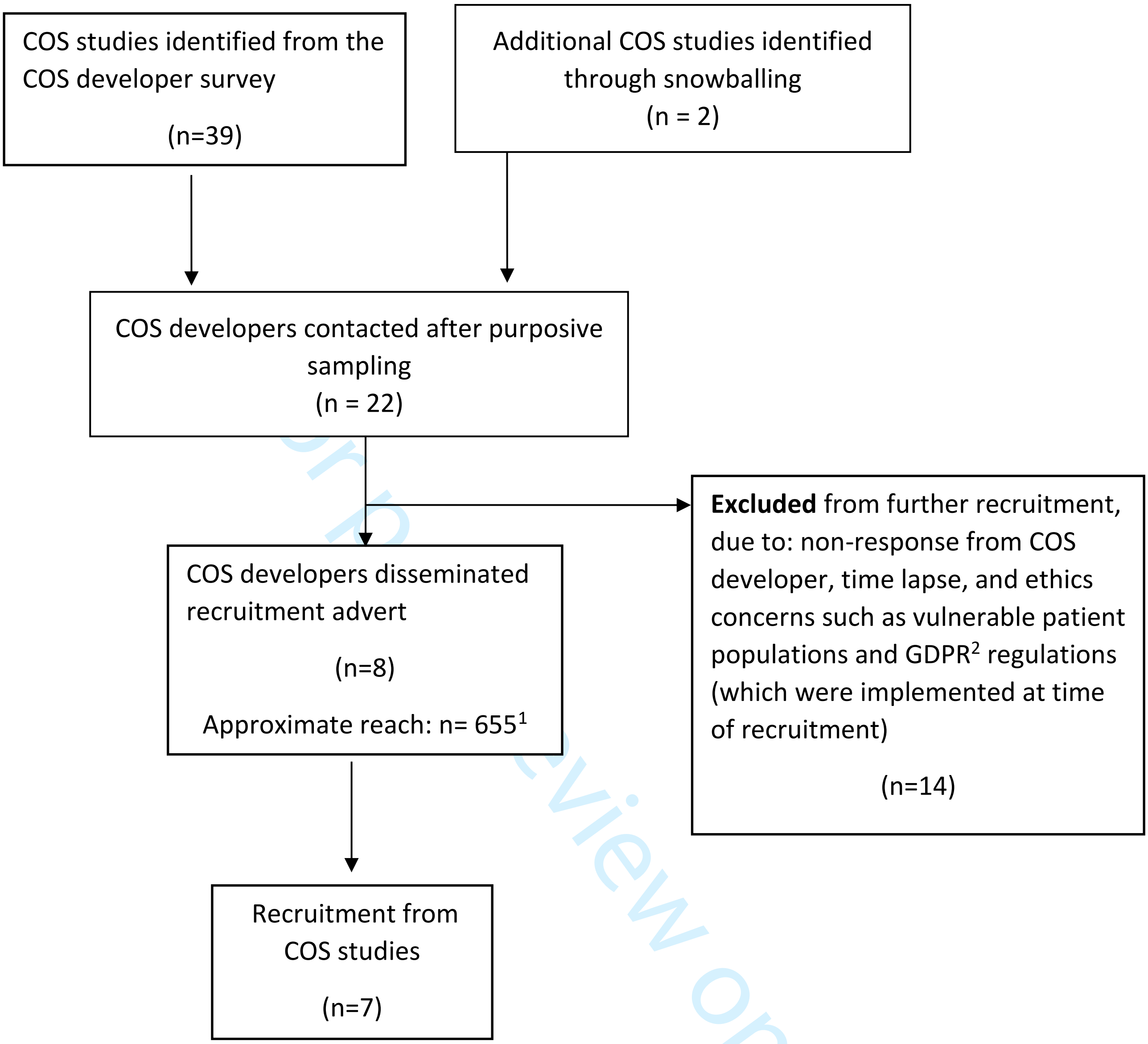

Figure 1: Sampling of COS studies that fit our sampling framework. [1]Reach of two COS studies is unknown, approximate relates to the other 6 COS studies. [2] General Data Protection Regulation (GDPR) is a European Union (EU) law regulation regarding data protection and privacy for all individuals within the EU and the European Economic Area [15]

For peer review only

Standards for Reporting Qualitative Research (SRQR)*

http://www.equator-network.org/reporting-guidelines/srqr/

Title and abstract

	Page/line no(s).
**Title** - Concise description of the nature and topic of the study Identifying the study as qualitative or indicating the approach (e.g., ethnography, grounded theory) or data collection methods (e.g., interview, focus group) is recommended	Pg. 1/ Line 2-4
**Abstract** - Summary of key elements of the study using the abstract format of the intended publication; typically includes background, purpose, methods, results, and conclusions	Pg.2/ Line 2-25

Introduction

**Problem formulation** - Description and significance of the problem/phenomenon studied; review of relevant theory and empirical work; problem statement	Pg.3/ Line 1-22
**Purpose or research question** - Purpose of the study and specific objectives or questions	Pg.3/ Line 23-30

Methods

**Qualitative approach and research paradigm** - Qualitative approach (e.g., ethnography, grounded theory, case study, phenomenology, narrative research) and guiding theory if appropriate; identifying the research paradigm (e.g., postpositivist, constructivist/ interpretivist) is also recommended; rationale**	Pg.4/ Line 3-5
**Researcher characteristics and reflexivity** - Researchers' characteristics that may influence the research, including personal attributes, qualifications/experience, relationship with participants, assumptions, and/or presuppositions; potential or actual interaction between researchers' characteristics and the research questions, approach, methods, results, and/or transferability	Pg.5/ Line 6-8
**Context** - Setting/site and salient contextual factors; rationale**	Pg.4 / Line 19-20
**Sampling strategy** - How and why research participants, documents, or events were selected; criteria for deciding when no further sampling was necessary (e.g., sampling saturation); rationale**	Pg.4 / Line 7-11<br>Pg. 4/ Line 41<br>Pg.5/ Line 1-4
**Ethical issues pertaining to human subjects** - Documentation of approval by an appropriate ethics review board and participant consent, or explanation for lack thereof; other confidentiality and data security issues	Pg.5/ Line 4-5
**Data collection methods** - Types of data collected; details of data collection procedures including (as appropriate) start and stop dates of data collection and analysis, iterative process, triangulation of sources/methods, and modification of procedures in response to evolving study findings; rationale**	Pg. 4/ Line 17-32

For peer review only - http://bmjopen.bmj.com/site/about/guidelines.xhtml

**Data collection instruments and technologies** - Description of instruments (e.g., interview guides, questionnaires) and devices (e.g., audio recorders) used for data collection; if/how the instrument(s) changed over the course of the study	Pg. 4 Line 17-32
**Units of study** - Number and relevant characteristics of participants, documents, or events included in the study; level of participation (could be reported in results)	Pg.6 /Line 1-15<br>Pg.7 / Line 25-33<br>Pg. 8/ Line 1-6
**Data processing** - Methods for processing data prior to and during analysis, including transcription, data entry, data management and security, verification of data integrity, data coding, and anonymization/de-identification of excerpts	Pg. 5/ Line 31-32
**Data analysis** - Process by which inferences, themes, etc., were identified and developed, including the researchers involved in data analysis; usually references a specific paradigm or approach; rationale**	Pg. 4/ Line 34-39
**Techniques to enhance trustworthiness** - Techniques to enhance trustworthiness and credibility of data analysis (e.g., member checking, audit trail, triangulation); rationale**	Pg. 4/ Line 34-41

Results/findings

**Synthesis and interpretation** - Main findings (e.g., interpretations, inferences, and themes); might include development of a theory or model, or integration with prior research or theory	Pg. 12-16 inclusive
**Links to empirical data** - Evidence (e.g., quotes, field notes, text excerpts, photographs) to substantiate analytic findings	Pg.12 -16 inclusive

Discussion

**Integration with prior work, implications, transferability, and contribution(s) to the field** - Short summary of main findings; explanation of how findings and conclusions connect to, support, elaborate on, or challenge conclusions of earlier scholarship; discussion of scope of application/generalizability; identification of unique contribution(s) to scholarship in a discipline or field	Pg.18/ Line 1-42<br>Pg.19 / Line 1-42<br>Pg. 20/ Line 1-16
**Limitations** - Trustworthiness and limitations of findings	Pg.20 /Line 22-27

Other

**Conflicts of interest** - Potential sources of influence or perceived influence on study conduct and conclusions; how these were managed	Pg. 21/ Line 14-16
**Funding** - Sources of funding and other support; role of funders in data collection, interpretation, and reporting	Pg. 21 /Line 21-24

*The authors created the SRQR by searching the literature to identify guidelines, reporting standards, and critical appraisal criteria for qualitative research; reviewing the reference lists of retrieved sources; and contacting experts to gain feedback. The SRQR aims to improve the transparency of all aspects of qualitative research by providing clear standards for reporting qualitative research.

For peer review only - http://bmjopen.bmj.com/site/about/guidelines.xhtml

**The rationale should briefly discuss the justification for choosing that theory, approach, method, or technique rather than other options available, the assumptions and limitations implicit in those choices, and how those choices influence study conclusions and transferability. As appropriate, the rationale for several items might be discussed together.

Reference:

O'Brien BC, Harris IB, Beckman TJ, Reed DA, Cook DA. **Standards for reporting qualitative research: a synthesis of recommendations.** *Academic Medicine*, Vol. 89, No. 9 / Sept 2014 DOI: 10.1097/ACM.0000000000000388

For peer review only

For peer review only - http://bmjopen.bmj.com/site/about/guidelines.xhtml

BMJ Open

Participating in core outcome set development via Delphi surveys: Qualitative interviews provide pointers to inform guidance

Journal:	*BMJ Open*
Manuscript ID	bmjopen-2019-032338.R1
Article Type:	Original research
Date Submitted by the Author:	18-Sep-2019
Complete List of Authors:	Biggane, Alice M; University of Liverpool, Biostatistics; INSERM, CRESS, INRA, Université de Paris<br>Williamson, Paula; University of Liverpool, Biostatistics<br>Ravaud, Philippe; INSERM, CRESS, INRA, Universite de Paris; Columbia University, Epidemiology<br>Young, Bridget; University of Liverpool, Health Services Research
**Primary Subject Heading**:	Research methods
Secondary Subject Heading:	Qualitative research
Keywords:	Delphi survey, patient participation, core outcome set development

SCHOLARONE™ Manuscripts

18/09/2019
BMJ Open submission

Title page

Title:

Participating in core outcome set development via Delphi surveys: Qualitative interviews provide pointers to inform guidance

Author details:

Alice M Biggane[1,2]*, Paula R Williamson[1], Philippe Ravaud[2,3,4], Bridget Young[5]
[1] Department of Biostatistics, University of Liverpool, Liverpool, UK.
[2]Université de Paris, CRESS, INSERM, INRA, F-75004 Paris, France
[3]Centre d'Épidémiologie Clinique, AP-HP (Assistance Publique des Hôpitaux de Paris) Hôpital Hôtel Dieu Paris Cedex 4, France
[4]Department of Epidemiology, Columbia University Mailman School of Public Health, New York, USA
[5]Department of Health Services Research, University of Liverpool, Liverpool, UK.

Abstract word count:

249

Manuscript Word count:

6252

Publication type:

Research article

Key words:

Delphi survey, patient participation, core outcome set development

Funding:

This project has received funding from the European Union's Horizon 2020 research and innovation programme under the Marie Sklodowska-Curie grant agreement No 676207.

Conflict of interests:

Paula Williamson chairs the Management Group of the COMET Initiative.
Bridget Young is a member of the COMET PoPPIE (People and Public Participation, Involvement and Engagement) Working Group.
No other authors have any competing interests.

Corresponding author details:

Alice M Biggane
Department of Biostatistics,
University of Liverpool,

For peer review only

For peer review only - http://bmjopen.bmj.com/site/about/guidelines.xhtml

18/09/2019
BMJ Open submission

Liverpool,
UK.
abiggane@liverpool.ac.uk

Abstract:

Objectives:

To explore participants' views of Delphi surveys in core outcome set (COS) development.

Study Design and Setting

Patients and health professionals (n=24) from seven recently concluded COS studies that had involved a Delphi survey took part in semi-structured qualitative interviews (telephone and email exchange). Interviews explored participants' understanding of COS and their experiences of the Delphi survey. Analysis was thematic.

Results:

Several interviewees had previously participated in two or more COS or Delphi surveys. Those with multiple experiences of participation generally understood the purpose of COS and were satisfied with the Delphi survey. However, some interviewees who were first-time participants struggled to understand the purpose of COS and aspects of the Delphi survey, which limited their contribution and satisfaction with the study. Interviewees also differed in how they interpreted and subsequently used the written documentation provided to COS participants. Some interviewees wanted guidance regarding whose perspective to take into account when scoring outcomes and on how to use the scoring system. Interviewees reported being motivated to take part by the international and expert consensus aspects of the Delphi survey. A few interviewees reported experiencing either positive or negative emotional impacts arising from when they reviewed outcomes and stakeholder feedback.

Conclusion:

This study identifies important information that should be communicated to COS Delphi study participants. It also indicates the importance of communicating about COS Delphi studies in ways that are accessible and salient to participants, to enhance their experience of participation and make the process more meaningful for all.

Strengths and Limitations

- This is the first study to explore, in-depth, the experiences of patients and health professionals who took part in core outcome set (COS) development via the Delphi survey

For peer review only - http://bmjopen.bmj.com/site/about/guidelines.xhtml

- A strength of this study is that we were able to ask interviewees specific, tailored questions thus exploring their personal perspectives and insights of COS Delphi study participation
- This study sampled an international selection of patients and health professionals
- This study sampled from COS Delphi studies in a range of health conditions
- Limitations include the retrospective nature of the interview

1. Introduction

Inconsistency in outcomes measured in clinical trials is a major concern across a multitude of health conditions, limiting the synthesis of available evidence and ability to reach reliable conclusions (1, 2).

Core outcome sets (COS) are one potential solution to this problem. A COS is a minimum set of agreed standardised outcomes which should be measured and reported in all trials in a specific condition as a minimum (3). Three important stakeholder groups in the development of COS for trials are health professionals, patients and those who will use the COS in research, such as clinical trialists or industry(4).

Several methods are used to include stakeholders as participants in COS development, including interviews, focus groups, nominal group technique and Delphi surveys. Delphi surveys, used singularly or in combination with other methods, are the most popular method of facilitating participation (5). These involve iterative rounds of questionnaires listing outcomes and asking participants to score the importance of each outcome. Scores are subsequently summarised across the various stakeholder groups and fed back to participants in the following round. This allows participants to consider the views of others before re-scoring each item. Furthermore, participants' views are anonymised which minimises the influence of power differentials between different stakeholders that can be problematic with direct communication between participants (6, 7). The creation, administration and analysis of Delphi surveys is relatively inexpensive. The availability of online Delphi survey platforms allows large samples and facilitates international development of COS, thus, ensuring they are relevant globally.

However, Delphi surveys have been described as potentially intimidating for some patient participants (7) and COS developers have acknowledged a need for guidance on conducting Delphi surveys and the consensus meetings which typically follow them (8). While recent surveys of COS participants indicate that their experiences of Delphi surveys have been generally favourable (9, 10), no research has explored in-depth the perspectives of patients and health professionals on participating in COS Delphi surveys. We therefore explored their opinions and experiences of participation to identify ways to enhance Delphi surveys for future participants in COS studies.

For peer review only - http://bmjopen.bmj.com/site/about/guidelines.xhtml

18/09/2019
BMJ Open submission

2. Methods

2.1 Research design

In the current study, EPITOME (Exploring Participant InpuT in Core Outcome Set DevelopMEnt), taking a broadly pragmatic approach we used semi-structured qualitative interviews to explore patients' and health professionals' experiences of participating in COS Delphi surveys.

2.2 Sampling strategies and recruitment

We used the responses of COS developers to a previous survey (5) to inform purposeful sampling of host COS studies from which to recruit interviewees. This survey was informed by searches of the COMET (Core Outcome Measures in Effectiveness Trials) Initiative database. COMET has created and maintains a publicly accessible database (www.comet-initiative.org) of planned, ongoing and completed COS projects and is updated annually with published studies that have been identified through a systematic review. The survey was sent to all COS developers who had published or registered a study with COMET since 2013.Host studies were eligible if they had involved a Delphi survey, had patient participants, included participants from more than one country, and had concluded no more than six months prior to the interview. COS developers of each host study distributed a recruitment advert (Supplementary File 1) to all stakeholders who registered for the first round of the Delphi survey. The advert invited interested individuals to contact AMB who provided a participant information sheet. For each host COS, we aimed to interview up to two patients and two health professionals. Interviewees were sent a thank you card and £15 (or currency equivalent) shopping voucher as an acknowledgement.

2.3 Data collection

Interviewees were geographically dispersed so were interviewed via telephone or email exchange. The data were collected between October 2017 and June 2018. At the time of interview, interviewees were between seven months and six weeks from having participated in the final round of the host COS Delphi. All telephone interviews were semi-structured and used a topic guideline which allowed for a conversational approach to be adopted to explore issues that we anticipated to be important, while enabling interviewees to raise areas that were important to them. COS developers and public contributors with experience of COS development informed the initial development of the topic guide (Supplementary File 2), as did previous qualitative research (11). Ongoing data analysis informed the further iterative development of the topic guide. Furthermore, the interviewer, AMB, tailored questions for each interviewee by reviewing available information on the host study prior to every interview. This information included, for example: participant information materials such as guidance sheets and videos, the number of rounds, scoring systems used, numbers of domains and outcomes scored and examples of outcomes scored. For one host study a screenshot of the Delphi survey was supplied by the developers which AMB then used as a memory aid with interviewees from that COS Delphi study. Email interviews followed a similar format asking a range of open-ended questions across topics, if necessary the interviewer, AMB followed up on responses with additional open-ended questions to further explore the interviewees' answers and comments. All interviewees gave informed consent. The first two

For peer review only - http://bmjopen.bmj.com/site/about/guidelines.xhtml

audio-recorded interviews were transcribed verbatim by AMB, the remainder were transcribed verbatim by a University of Liverpool approved transcription agency into Microsoft Word. Transcripts were checked and anonymised before being analysed. The data is currently held in password encrypted files on The University of Liverpool's secure server. AMB, who was a PhD student supervised by PRW and BY, conducted all interviews in English. Before starting data collection, she received training in qualitative methods.

2.4 Data analysis

Data analysis drew on Braun and Clarke's six phase thematic approach (12). Analysis was initially deductive following the topic guides but became more inductive as the analysis progressed (12) and ranged from line-by-line coding, to considering whole transcripts. AMB initially read the transcripts and reflective fieldnotes that she had made immediately after each interview to inform her interpretations. A codebook was developed for the content using open coding. By grouping the codes together, recurring patterns and themes were identified and organised into categories (12). AMB led the analysis, which she periodically discussed with BY and PRW, who each read a sample of the transcripts and reviewed reports of the developing analysis. All three agreed that data saturation (the point at which new data cease to contribute to the analysis) had been reached after twenty-four interviews. Microsoft Word was used to facilitate coding and analysis (13).

2.5 Ethics

While accepting that quality procedures cannot promise quality (14), the reporting of this study was informed by relevant guidance (15). The study was approved by the University of Liverpool ethics committee (reference: 1969). It is important to note the authors' interests in COS development, further details of which can be found in the "Declarations" section of this manuscript, will have inevitably shaped the study and its findings.

2.6 Patient and public involvement statement

Patients and the public were involved in developing and reviewing the topic guide, recruitment advert and participant information sheets used in this study.

2.7 Definitions

We use the term 'patient' to refer to patients, carers, service users and people from organisations who represent these groups. We use 'health professional' to refer to clinicians and pharmacists. Interview excerpts shown below were selected to demonstrate the findings and our interpretations. Health professionals are indicated by "HP" and patients by "P", the COS in which they took part is indicated by "COS" and a number e.g. HP1COS1; "[…….]" indicates text removed for succinctness.

For peer review only - http://bmjopen.bmj.com/site/about/guidelines.xhtml

18/09/2019
BMJ Open submission

3. Results

3.1 COS study sampling and interviewee characteristics

We initially identified 39 potential host COS studies via the survey (5) (Figure 1). Two further ongoing COS studies were brought to our attention by COS developers, which were not in the COMET database at the time of the survey, but were subsequently added. We contacted the developers of 20 of these COS studies in batches to inform purposive sampling to achieve maximum variation. Of these 20, we excluded 14 studies from further consideration (Figure 1). We distributed our recruitment advert, via the COS developers, to the participants in the remaining six COS studies, plus the two further studies brought to our attention, giving eight unique online COS studies. Of these we recruited participants from seven COS studies. These studies covered: geriatrics (COS1), dermatology (COS2), other (COS3), cancer (COS4), paediatrics (COS5), gynaecology and obstetrics (COS6) and otorhinolaryngology (COS7), and all aimed to recruit international participants.

They varied in terms of the number of outcomes to be scored, the number of rounds, scoring system, and in the ways feedback was presented to Delphi survey participants.

Following distribution of our advert, forty participants from the seven COS studies contacted us. We did not take forward interviews with 11 of these (6 health professionals, 2 patients and 3 unknown status) as interview quotas for their COS study had been reached. Of the 29 participants invited for interview, 24 participated. Of the remaining five, two patients withdrew as they were unable to recall any details of their COS study whilst two patients and a health professional did not respond after the initial contact.

Table 1 summarises the demographic characteristics of the 24 interviewees, two interviews were completed by email exchange, and the remainder were telephone interviews. Twelve (50%) were resident in the United Kingdom (UK), four in Ireland, three in Canada, and one from each of Australia, Italy, Singapore, Spain and the Netherlands. Twenty-two interviewees described themselves as having professional occupations, two patient interviewees were retired and did not disclose their most recent occupation. Ten interviewees (three patients and seven health professionals) had previous experience of COS, Delphi surveys or both. One of the three patients with previous experience was also the patient research partner (involved in the design and conduct) of the COS development about which they were interviewed.

For peer review only - http://bmjopen.bmj.com/site/about/guidelines.xhtml

18/09/2019
BMJ Open submission

Identifier	Gender:	Age range (years):	Country:	Prior participatory experience:	
				COS development	Delphi survey
P1COS1	Male	65-74	UK	No	No
P2COS1	Female	≥75	UK	No	No
P3COS2	Female	35-44	UK	No	No
P4COS3	Female	Undisclosed	Canada	Yes	Yes
P5COS2	Male	45-54	UK	No	No
P6COS3	Female	55-64	Canada	Yes	Yes
P7COS4	Female	55-64	UK	No	No
P8COS4	Female	55-64	Netherlands	No	No
P9COS5	Female	35-44	Ireland	No	No
P10COS6	Female	45-54	Ireland	Yes[a]	Yes
P11COS7	Male	55-64	UK	No	No
P12COS7	Female	65-74	UK	No	No
P13COS2	Female	55-64	UK	No	No
HP1COS1	Female	45-54	Canada	No	Yes
HP3COS4	Male	45-54	Spain	Yes	Yes
HP4COS2	Female	35-44	Singapore	Yes	Yes
HP5COS4	Male	35-44	UK	Yes	Yes
HP6COS5	Female	55-64	UK	No[b]	No
HP7COS5	Female	25-34	Ireland	No[c]	No
HP8COS5	Female	35-44	UK	No[b]	No
HP9COS5	Female	65-74	Ireland	Yes	Yes
HP10COS6	Female	35-44	Italy	No	No

For peer review only - http://bmjopen.bmj.com/site/about/guidelines.xhtml

HP11COS6	Male	65-74	UK	Yes	Yes
HP12COS6	Female	55-64	Australia	Yes	Yes

Table 1: Interviewee demographic characteristics. [a] Interviewee was also the patient research partner of the COS study they were interviewed in relation to. [b] Two health professionals stated awareness/knowledge of COS and Delphi survey but had not participated previously. [c] One health professional was involved in an earlier phase of the COS study for which they participated in the Delphi survey.

For peer review only

3.2 Findings from interviews

For most interviewees, taking part in an online Delphi survey several months ago had not been a particularly salient or memorable event. Therefore, some interviewees, particularly patients, at times struggled to recall details of the host COS and so the interviewer had to provide them with brief prompts or reminders throughout the interviews. For example, P9COS5 had *“signed up to a lot of studies”* during the same time period, and asked the interviewer to remind her of what the study was about. On explaining the topic of the Delphi survey and giving some reminders of the process such as the number of rounds and the process of reviewing and scoring outcomes, P9COS5 commented that she could recall filling out only one round of the Delphi survey. Thus her interview is in relation to that only.

While all participants in each of the seven COS studies had access to resources such as information sheets (and to online videos for two of COS studies), which explained the purpose and format of the study, interviewees differed in how accurately and fully they understood the purpose of COS and the process of the Delphi survey.

3.3 Synthesis and interpretation

In what follows we present five thematic findings from our interviews as follows: i) how previous experience helped interviewees understand COS Delphi studies, ii) the differences in how participants understand the processes and purposes of Delphi surveys, iii) the question of who is being represented in the COS Delphi studies, iv) the motivational and emotional aspects of COS Delphi participation and v) how the scoring system used in Delphi surveys are understood by participants

3.3.1 Previous experience helped interviewees understand COS Delphi studies

As indicated in Table 1 several interviewees had previous experience of COS and Delphi surveys. In comparison to those without such experience, these interviewees generally showed a better understanding of the purpose of COS and indicated greater satisfaction with the Delphi survey. HPs with previous experience (n=7) praised COS for their importance and usefulness in research, and the Delphi survey method for its simplicity. HP5COS4 said *“that’s the beauty of it, it is just not a difficult, all the hard work is done by the people that analyse the data. It is just like answering a customer service survey from Sky isn’t it? Click next, next, next you just do it don’t you, but I would put more effort to this than I would do a customer survey from Sky because it is more important to me.”*

HPs without previous experience talked about having about read up to COS and Delphi surveys or of seeking advice from colleagues and peers to enhance their understanding of the study and prepare for their participation. For example, HP7COS5 took part in an earlier event for the same COS study at which the developers had been present; *“it made me think more fully about the bigger picture of research going forward and how these processes like the Delphi survey feed into that”* and that otherwise she *“would have approached it in a less informed way.”*

Three patient interviewees also spoke about the impact of their previous experiences in COS Delphi studies. Over the course of these studies, they described their experience evolving from one of confusion during their first study to one of enjoying the process and better

For peer review only - http://bmjopen.bmj.com/site/about/guidelines.xhtml

understanding the purpose of COS with each subsequent study "*once you get the hang of it, I really enjoy doing them because I like where it takes you*" (P6COS3). P10COS6 spoke of not having a *"bull's notion what is going on"* in earlier studies with regard to both the purpose and method of COS development and had *"to do a lot of online research myself to learn"*, despite receiving information sheets for each study. Reflecting on this evolving experience of COS and Delphi surveys during her interview, she suggested that providing participants with a visual synopsis of the purpose of COS and Delphi survey method from the outset of a study would be helpful: *"I would have assimilated the message much quicker."*

Patient interviewees (n=9) with no previous experience, varied in their understanding of the purpose of the Delphi survey. The comments of some showed that they understood the Delphi survey's purpose was to reach consensus on which core outcomes to include. For example, P7COS4 explained the study was: *"looking at how people felt with their recovery […] what they went through and what they were left with and how important those were to the person involved*." In contrast, others such as P8COS4 described the Delphi survey's purpose more vaguely as to gather a *"broad base of information on how many different people experience the treatment."* Moreover, she did not talk about the process in terms of prioritising the outcomes listed or reaching consensus amongst stakeholders. P1COS1 was confused about whether his study was complete or if he should expect further rounds of the survey: *"I don't even know that you could say a line had been drawn under it*." P11COS7 reflected on whether he *"could have done more to understand how the process worked earlier on. Particularly with the […] expert involvement, I now understand so next time I shall be even better at it"* and suggested *"a practice run"* would have been useful before entering the actual study. In a few cases participants indicated that their lack of understanding had influenced their overall experience of participation, *"I think one of my real concerns is that I didn't really contribute anything to the research because I really wasn't sure what I was doing"* (P2COS1).

3.3.2 Helping participants understand the purpose and process of Delphi surveys - one size does not fit all

The findings indicate that interviewees had different needs for support to aid their understanding of the purpose and process of COS Delphi surveys. P3CSO2 and P5COS2 were two first-time patient participants. They both received the same study documentation and said they reviewed it. However, their accounts indicated that they differed in their understanding of the documentation, and these differences influenced their contributions to and experiences of the study.

P5COS2 thought the study documentation he received was *"appropriate"*, elaborating *"I have worked in the past in IT, in pharmaceuticals, in politics[…]so I am quite happy to see text that is fairly technical in nature or fairly clinical in nature and you know that is something I find easy enough to get to grips with."* He thought that the study "*was a very constructive thing to do. And I could see personally, something like that being done prior to any clinical trial, so that the end points of the clinical trial […] look at, you know how beneficial say a product is from the patient's perspective*."

For peer review only - http://bmjopen.bmj.com/site/about/guidelines.xhtml

In contrast, P3COS2 who worked in marketing commented that she *"didn't understand the terminology"* in the documents and as a result described being *"switched off from the process element […] psychologically I was just focussed on taking part and having my say."* She wondered if the study and its data would get *"stored away somewhere in a filing cabinet and forgotten about [….] I think what was lacking in the communication is how this is going to actually practically inform future research. And maybe that is my lack of understanding of how these sort of surveys work, and how these outcome surveys work, I don't really get, how that will translate into future treatments."* In response to P3COS2's comment, the interviewer explained that COS were used as minimum sets of outcomes in clinical trials so that evidence can be compared across studies and inform decision making regarding treatments. The interviewer added that the Delphi survey was a method to develop the COS by seeking consensus amongst relevant experts including patients. In response, P3 recalled that she had received information to that effect in the study documentation before adding *"I really wish that had been captured in the communication a bit more clearly […] maybe I'd have done things differently*."

For peer review only

For peer review only - http://bmjopen.bmj.com/site/about/guidelines.xhtml

18/09/2019
BMJ Open submission

3.3.3 Representation in the Delphi survey- who and when

Both HP and patient interviewees raised the issue of "who they should be representing?" when completing the Delphi survey. They questioned whether they should try to think or imagine what outcomes fellow patients or HPs would likely prioritise when scoring the outcomes study, or whether they should focus only on their own opinions and priorities. None reported receiving guidance on this.

P5COS2 thought *"it can only be a genuine result if everybody says what they personally feel"* and "*trying to guess* [how others feel]*"* would defeat the objective. This contrasts with P7COS4, a female who described trying to answer the outcomes section of that was applicable to males only: "*I just thought well if I was in that situation I will answer it as if I was that person maybe you know. […] Yes maybe I shouldn't have done that."*

In COS3, both patient interviewees were also advocates in a relevant patient organisation, and both had previous experience of COS Delphi studies. P6 described how she *"learned very early on"* to answer from her own perspective. Conversely, P4 drew on her knowledge of the perspectives of other patients from discussions she had had through her work with the patient organisation *"I do try to work in their concerns and the issues that they have."* She added that COS developers should consider how the different phases in a patient's journey and their life could affect the way they scored outcomes: *"my priorities are different now, than they were when I was diagnosed over 30 years ago […] you know different things would have affected me. […] over the years with the chronic disease you learn to live with it and adapt to it, so […] yes I think that can affect your responses too."*

HPs touched on similar issues regarding who to represent when scoring outcomes, although compared to patients, this was less prominent in their accounts. HP1COS1, was an academic, a service provider and a policy maker. Referring to both her experiences as a professional and her personal opinions, she explained that she drew on *"a bit of both"* when scoring outcomes. Similarly, HP11COS6, an academic and service provider, explained *"it was a mixture of, of relating it to myself and relating it to patients. But I was, even when I was relating it to myself I was relating it to me thinking of myself as a patient or the father of a patient or something like that."*

For peer review only - http://bmjopen.bmj.com/site/about/guidelines.xhtml

18/09/2019
BMJ Open submission

3.3.4 Motivational and emotional aspects of participation

A few patients and HPs talked about the motivational and emotional aspects of their participation.

Health professionals praised the Delphi survey method of COS development for its consensual and collaborative approach, and cited the opportunity to learn from international colleagues as one of the motivations for participating. They also spoke of their belief in the importance of COS in their field and their desire to contribute.

Patients described being "happy" that they could contribute their experiential knowledge and have input in research studies relevant to them. Some saw the COS study as one of the few research projects relevant to their condition and this was a motivating factor in their participation.P8COS4 talked about how her illness was *"rare"* and how information and research on the illness was limited *"so it was great for us* (other patients) *and for me specifically you know to fill in something that was specifically to do with my* (illness)", she further elaborated that the COS study *"made us feel someone was listening or someone was going to help us."* P3COS2 talked about how she felt *"happy"* to be included in research relevant to her, as she was outside the age range that was typical for patients with the health condition concerned. Similarly, P5COS2 *"thought it was quite exciting the fact that they would ask regular kind of sufferers of particular problems what do you think should be included in a trial. What outcomes do you think are important and everything and getting feedback from people outside the scientific community. I thought was quite cool and as somebody who suffers from various medical conditions the ability for me to give my input on what I think is important to a patient."*

P6CO3 had participated in multiple COS Delphi studies. She described her enthusiasm for the Delphi survey as a motivation to participate: "*every time I do them, I enjoy them more I really, really like the process"* and her willingness to participate in studies that used the resulting COS: *"you might have a preconceived notion of what something should be, or perspective on what something should be, or what the final product should look like, and it takes you in a different direction and if you just kind of you know let go and let it take you where it takes you through the questions and the feedback and everything I think it is a really interesting way of coming up with a list and I think it is a really true list."*

Two patients and one health professional indicated that reviewing the list of outcomes had affected them emotionally. Speaking of when she reviewed the scores provided by fellow participants in the second round of P8COS4 commented that she had: *"changed some of my answers on the second round, when I was thinking about having a possible* (intervention removed) *then I was like oh, I wouldn't want that at all […] I was sort of realising that I was grateful for where I was basically."* HP7COS5 said that when reviewing the fellow participants' feedback *"there were definitely moments of almost insecurity I suppose because you are aware, […] you are in amongst a group of other people who are very familiar with this field and experts […]"* She described initially feeling uncertain about her answers: *"it is ok to obviously be encouraged to check back on yourself and to be really thoughtful when you are kind of giving those sorts of answers [….] so I think there was a little bit of both an awareness*

For peer review only - http://bmjopen.bmj.com/site/about/guidelines.xhtml

of needing to stay objective but there was certainly a more subjective, emotive aspect to seeing how other people were answering."

P2COS1 spoke of how reviewing the outcomes as part of the COS study had made her aware of outcomes that she had not previously realised were associated with her condition and treatment: *"A lot of the outcomes I would never have thought of those as outcomes from the sort of medication I am on if you see what I mean."* She described how this had affected her: *"I am seriously worried about that. […] I was given no indication* [by healthcare provider] *[…] that I need to be careful."*

For peer review only

For peer review only - http://bmjopen.bmj.com/site/about/guidelines.xhtml

3.3.5 Scoring system

The scoring systems in the seven host COS studies used either a 9- (n=6) or 5- (n=1) point Likert scale. In five of the COS that used a 9-point Likert, scores were further differentiated as: 1-3 'Not important' (n=4) or 'Limited importance' (n=1), 4-6 'Important but not critical', 7-9 'Critical'. In the sixth, the anchor descriptions were 'not at all important' (1) and 'extremely important' (9). In the COS that used a 5-point Likert scale, participants were asked to rate their level of agreement on a series of statements regarding potential outcomes, with scores labelled: 1 Strongly disagree, 2 Disagree, 3 Ambivalent, 4 Agree, 5 Strongly agree.

Several interviewees did not comment on the scoring system during their interview. Those who did comment varied from praising or indicating satisfaction with the scoring system, to wanting a system with fewer categories and further guidance on how to apply the scale, although the majority of interviewees were positive about the scales used in COS Delphi studies that they had taken part in. Those who expressed satisfaction with the 9-point scales, indicated that they were familiar with using these: *"I am usually happy with Likert scales so, fine"* (HP12COS6), while another interviewee summed up her experience of the scales as *"not a big deal"* (P4COS3).

Interviewees who took part in a COS that used a 9-point scale and liked it praised the wide range of options and the three distinct bands as helpful. For example HP9COS5 commented *"I liked the way they set it out in that they were, you know while it was 9 it was important, not so important and least important so that even within those categories one could actually subdivide them, and I actually think I liked that. Sometimes you know you are asked you know, should something be important, and there are kind of gradations within importance, and so I think that for me I liked that subdivision. It gave me a little bit more flexibility."* P7COS4 noted *"grading it you know, systematically up from 1 to 9 so yes that was useful because it give you, although a lot of my scores were up on the higher range there were a couple of lower ones so I think the having 1 to 9 was a good idea."*

Other interviewees had a preference for fewer categories. Speaking of the 9-point scale in her study, P2COS1 commented "*I really don't think a score from 1 to 10 is realistic. […] maybe if you are a very skilled researcher yourself you might be able to deal in that level of gradation but I don't think the vast majority of us can. I think, you know, a 5 point rating scale is the most that most of us could do. You know with any degree of accuracy."* Similarly, also speaking of the 9 point scale HP8COS5 said "*what is the difference between a 6 and 7, you know what I mean if it is just sort of all in the middle of the road […] so whether or not it could have been less numbers to help make a more definitive answer."* However, like other interviewees who had a preference for a scale with fewer categories she acknowledged *"there might be reasonings behind why you have got 0-9 and that type of thing*." While some interviewees found the three bands on the 9-point scale helpful, responses from some health professionals and patients indicated that further guidance and support is needed to help them use the 9-point scale. Similarly P11COS7, a first time patient participant, raised the difficulties he experienced in *"connecting physical sensations with a numerical value"* when relating his physical symptoms to scoring outcomes. He added that this *"produces a certain anxiety between whether you pick 5, 6 or 7."*

For peer review only - http://bmjopen.bmj.com/site/about/guidelines.xhtml

HP6COS5 was the only interviewee who compared the scoring system to other methods of prioritisation when she flagged her overall preference for a numerical scale when scoring a long list of items in comparison to ranking them *"if I had been given the list and said you know rate these 1 to 20 it would have been harder to do."*

For peer review only

18/09/2019
BMJ Open submission

4. Discussion

4.1 Summary of findings

We found that while some interviewees understood the purpose of COS and the Delphi survey, others struggled to understand the purpose and aspects of the Delphi survey method which in turn influenced their contribution and experience of the study. The accounts of the interviewees indicate that COS participants would benefit from further guidance and support.

Interviewees could be broadly separated into two categories; those with and without previous experience of COS development and/or Delphi surveys. The accounts of those with previous experience, both health professionals and patients, showed they had a good understanding of the purpose of COS and were satisfied with the Delphi survey as a method of participation. Health professionals without previous experience reported engaging with relevant literature and colleagues prior to and during participation, thus enhancing their understanding and experience. In contrast, the accounts of patients without previous experience indicated considerable variation with some showing good understanding, while others understood little of the study and its purpose. Aspects that the latter group struggled with included understanding that the Delphi survey aimed to achieve consensus amongst stakeholders, applying the scoring system and knowing whose views to represent when participating. This limited their engagement and interpretation of the documentation they had received from COS developers, and their input and experience of COS development.

The importance of representing of all relevant stakeholder groups including patients in COS development (4, 7) is increasingly recognised, as it is in wider health research (16-18). There is also growing appreciation of the importance of supporting their participation in ways that are meaningful, thus avoiding tokenism and enhancing the credibility and validity of the resulting research (19, 20). However, our findings suggest that not all the interviewees thought their participation in COS development was meaningful, as the purpose and process of the study was communicated in ways that were not accessible for them. Theory surrounding health literacy describes its role in patient empowerment and advocates for information to be made accessible to all patients in appropriate formats (21-24) This is particularly important for patient participants in COS development, most of whom will not have taken part in this type of research previously nor have access to the literature or colleagues to illuminate the process. A few patient interviewees in this study indicated that they saw understanding COS Delphi studies as their personal responsibility or felt uncomfortable with their limited of understanding. However, when asking patients to participate in COS studies developers are inviting them to the world of research (7), thus, it is the responsibility of the COS development community to ensure the guidance and support is in place to allow meaningful participation. There has been a rapid expansion in the number of COS being developed, with an associated rapid increase in the number including patients in Delphi surveys. Our findings indicate that this expansion has perhaps outpaced the development of relevant guidance for Delphi studies to enable meaningful participation for all.

For peer review only - http://bmjopen.bmj.com/site/about/guidelines.xhtml

18/09/2019
BMJ Open submission

This study points to specific areas where further guidance and support is required to communicate the purpose of COS and the process of the Delphi survey, which we summarise as pointers for COS developers to consider in Table 2. This complements the findings of two recent surveys of COS Delphi study participants which indicated that they benefit from repeated guidance on principles of core outcome set development during the rounds, that reminders about these principles were acceptable (10), and that recruitment and retention of participants is more likely with personalised communication (9). To date the most common way of providing participant information regarding a research project is via written documentation. Much research has indicated poor health literacy is prevalent (25-28), thus the importance of ensuring plain language communication cannot be underestimated. However, this study's findings suggest not only is plain language communication required, but also further consideration of how to explain the purpose of COS in ways that are relatable and salient to patients. This explanation and delivery could make use of visual, written and auditory methods, such as analogies, infographics, visual metaphors, digital stories and other narrative forms. The most appropriate method or combination of methods is likely to depend on the population and health condition to which the COS will be relevant. The use of visual resources have been documented in other healthcare areas such as health promotion (29), patient education (30) and nursing training (31). In COS development demonstration videos of the Delphi survey enhanced participant retention to the study (9). The COMET website provides resources to help developers facilitate participation, including documents explaining COS in plain English and an animation video (http://www.comet-initiative.org/resources/PlainLanguageSummary), co-produced with members of the public.

This study also indicates areas in which further research and direction would be useful. The issues raised by interviewees regarding how to apply the scoring system, point to the need for better communication. The 9-point Likert scoring system where items are graded in accordance to their level of importance is a common method, recommended by the Grading of Recommendations Assessment, Development and Evaluation (GRADE) Working Group (32). There are statistical considerations in support of using a longer scale including the ability to calculate variance in scores. Thus, it is important that participants in COS Delphi studies have the information and support they need to apply this system. Involving patients and members of the public as active research partners would provide a patient perspective on the suitability of different aspects of the COS study from design to conclusion, including helping with the development of appropriate documentation, resources and support (7, 9).

Interviewees also raised the issue of whose perspective to take into account when scoring outcomes. Pending further research, we would recommend that in the first round of the Delphi survey COS developers ask participants to score according to their own individual perspective, not score according to the perspective of others. In the second or subsequent rounds participants should be asked to reflect on the scores of other participants, while being clear that they do not have to change their own scores. Having reflected participants should be asked to score according to their current view of what a COS in that specified health condition should include (33). Participants can be encouraged to score outcomes they have no experience of to date, but may experience in the future, although an "unable to score"

For peer review only - http://bmjopen.bmj.com/site/about/guidelines.xhtml

option or equivalent should also be provided for each outcome. A key exception to participants scoring from their own individual perspective is when carers act as proxy respondents in COS studies. In health research on certain patient populations there is often no alternative to using proxies (34, 35), yet there is evidence of discrepancies in how proxies prioritise outcomes compared to patients themselves (36, 37). During the first round of COS Delphi studies proxies should score according to what they anticipate is the perspective of the patient and not from their own perspective as a carer, and follow the same advice as other participants in subsequent rounds. Thus, COS developers should consider which proxies can provide a valid opinion on the anticipated perspective of the patient and how best to support this type of participation.

Some interviewees described the motivation and emotions associated with their participation. Understanding that participants are motivated to engage in COS development out of desire to contribute to the research topic and satisfaction with the Delphi survey's collaborative and international approach will be useful to COS developers when advertising and recruiting participants to their study. The emotional impact of participation requires consideration from developers and researchers when designing and conducting their COS studies to optimise the experience of participants and minimise any negative impacts on them.

**Pointers**
• COS developers should consider the most appropriate medium(s) to communicate their COS Delphi studies information and guidance<br><br>*Points to consider: Language used, target audience, health condition*<br><br>• COS developers need to ensure that the scoring system used is explained in ways that participants can understand.<br><br>• COS developers should explain to participants whose perspectives they should consider when scoring in different rounds<br><br>• COS developers should explain to participants that in the first round of the Delphi survey they should score outcomes according to their own individual perspective.<br><br>*Proxies: In the first round, COS developers should ask proxies to score according to what they anticipate is the perspective of the patient and not from their own perspective as a carer*<br><br>• COS developers should ask participants in second or subsequent rounds to reflect on the scores of other participants, while also being clear that participants do not have to change their own scores.<br><br>*Proxies: should follow the same advice as other participants in second or subsequent rounds*

For peer review only - http://bmjopen.bmj.com/site/about/guidelines.xhtml

- COS developers can encourage participants to score outcomes they have no experience of to date, but may experience in the future, although an "unable to score" option or equivalent should also be provided for each outcome.
- COS developers should consider the potential influence of their COS Delphi on participants and take appropriate steps to minimise negative effects.
- By understanding what motivates participants into COS Delphi studies, COS developers can devise appropriate recruitment and retention strategies

Table 2: Summary of the pointers and recommendations COS developers should consider when designing and conducting their COS Delphi studies

4.2. Strengths and weaknesses of the study

This study has provided insights into COS development via Delphi surveys from the perspective of participants. As previously noted, participation in the COS Delphi studies was not a particularly salient event for interviewees, however during their interviews they were provided with tailored prompts and reminders as needed.

This study only describes the experiences of participants who agreed to be interviewed, recruited from seven COS studies and limited to English-speakers. Those interviewed, including patients, mostly described themselves as having "professional backgrounds". Thus, while saturation was reached within our sample we note that interviewees' experiences and perspectives may not but typical of the wider patient population. However, by purposively sampling across a range of COS studies, we anticipate that our findings will be broadly transferable to other COS studies. Moreover, our interviewees were international, reflecting the increasing international development of COS.

5. Conclusion

This study's findings contribute to the growing evidence base on participation in COS development. The identification of areas where participants need enhanced guidance and support will be useful to future COS developers when planning their studies, enabling them to recruit and support participants towards a meaningful and positive experience of COS Delphi studies.

For peer review only - http://bmjopen.bmj.com/site/about/guidelines.xhtml

18/09/2019
BMJ Open submission

Declarations

Ethics approval and consent to participate

Ethical approval was granted from Health and Life Sciences Committee on Research Ethics (Human participants, tissues and databases) at The University of Liverpool, on 22/06/2017 (reference 1969). All interviewees were provided with full written information prior to interview commencement. All interviewees gave audio-recorded or written informed consent prior to commencing the interview and were free to end the interview at any time without providing a reason.

Consent for publication

Not applicable

Availability of data and materials

No additional data available

Competing interests

PRW helped to found the Core Outcome Measures in Effectiveness Trials (COMET) Initiative in 2010, which promotes the development and application of COS. COMET developed software, DelphiManager, for conducting online Delphi surveys.

BY has interests in the development of COS, since 2015 she has co-chaired the COMET People and Patient Participation Involvement and Engagement (PoPPIE) Working Group.

No other authors have any competing interests.

Funding

This project is a part of a MiRoR (Methods in Research on Research) funded PhD, which is being undertaken by AB. MiRoR has received funding from the European Union's Horizon 2020 research and innovation programme under the Marie Sklodowska-Curie grant agreement No 676207.

Authors' contributions

All authors have read and approved the final version of this manuscript. AB was the lead researcher on this project and was responsible for the preparation and drafting of the protocol, data collection and analysis and writing of this manuscript. PRW is a co-investigator and contributed to project conception, design, protocol writing, analysis, writing and proofreading of this manuscript. PR is a co-investigator and contributed to the methodology used in creating the sampling framework. BY is principal investigator on this project and is responsible for its design, protocol writing, analysis, writing and proofreading of this manuscript.

For peer review only - http://bmjopen.bmj.com/site/about/guidelines.xhtml

Acknowledgements

The authors would like to thank the COS developers and the interviewees for their time and input. The authors would like to acknowledge the Methods in Research on Research (MiRoR) network for their support and guidance, in particular Van Thu Nguyen, Ketevan Glonti and Melissa Sharp. They authors are also grateful to Camila Olarte Parra and Els Goetghebeur for their thoughtful comments and suggestions on the final draft of the manuscript.

For peer review only

For peer review only - http://bmjopen.bmj.com/site/about/guidelines.xhtml

18/09/2019
BMJ Open submission

References

1. Chalmers I, Glasziou P. Avoidable waste in the production and reporting of research evidence. Lancet. 2009;374: 10.1016/s0140-6736(09)60329-9
2. Chong LS, Sautenet B, Tong A, Hanson CS, Samuel S, Zappitelli M, et al. Range and Heterogeneity of Outcomes in Randomized Trials of Pediatric Chronic Kidney Disease. The Journal of Pediatrics. 2017;186:110-7. e11:
3. Williamson PR, Altman DG, Blazeby JM, Clarke M, Devane D, Gargon E, et al. Developing core outcome sets for clinical trials: issues to consider. Trials. 2012;13:132: 10.1186/1745-6215-13-132
4. Kirkham JJ, Davis K, Altman DG, Blazeby JM, Clarke M, Tunis S, et al. Core Outcome Set-STAndards for Development: The COS-STAD recommendations. PLoS Medicine. 2017;14(11):e1002447:
5. Biggane AM, Brading L, Ravaud P, Young B, Williamson PR. Survey indicated that core outcome set development is increasingly including patients, being conducted internationally and using Delphi surveys. Trials. 2018;19(1):113:
6. Sinha IP, Smyth RL, Williamson PR. Using the Delphi technique to determine which outcomes to measure in clinical trials: recommendations for the future based on a systematic review of existing studies. PLoS Medicine. 2011;8(1):e1000393:
7. Young B, Bagley H. Including patients in core outcome set development: issues to consider based on three workshops with around 100 international delegates. Research Involvement and Engagement. 2016;2(1):25:
8. Gargon E, Williamson PR, Young B. Improving core outcome set development: qualitative interviews with developers provided pointers to inform guidance. Journal of Clinical Epidemiology. 2017;86:140-52:
9. Hall DA, Smith H, Heffernan E, Fackrell K. Recruiting and retaining participants in e-Delphi surveys for core outcome set development: Evaluating the COMiT'ID study. PloS ONE. 2018;13(7):e0201378:
10. Turnbull AE, Dinglas VD, Friedman LA, Chessare CM, Sepúlveda KA, Bingham III CO, et al. A survey of Delphi panelists after core outcome set development revealed positive feedback and methods to facilitate panel member participation. Journal of Clinical Epidemiology. 2018;102:99-106:
11. Ritchie J, Lewis J, Nicholls CM, Ormston R. Qualitative research practice: A guide for social science students and researchers: Sage; 2013.
12. Braun V, Clarke V, Terry G. Thematic analysis. Qual Res Clin Health Psychol. 2014;24:95-114:
13. La Pelle N. Simplifying qualitative data analysis using general purpose software tools. Field Methods. 2004;16(1):85-108:
14. Barbour RS. Checklists for improving rigour in qualitative research: a case of the tail wagging the dog? BMJ: British Medical Journal. 2001;322(7294):1115:
15. O'Brien BC, Harris IB, Beckman TJ, Reed DA, Cook DA. Standards for reporting qualitative research: a synthesis of recommendations. Academic medicine : journal of the Association of American Medical Colleges. 2014;89(9):1245-51: 10.1097/acm.0000000000000388
16. Boote J, Baird W, Beecroft C. Public involvement at the design stage of primary health research: a narrative review of case examples. Health policy (Amsterdam, Netherlands). 2010;95(1):10-23: 10.1016/j.healthpol.2009.11.007
17. Ocloo J, Matthews R. From tokenism to empowerment: progressing patient and public involvement in healthcare improvement. BMJ Qual Saf. 2016;25(8):626-32:
18. Supple D, Roberts A, Hudson V, Masefield S, Fitch N, Rahmen M, et al. From tokenism to meaningful engagement: best practices in patient involvement in an EU project. Research Involvement and Engagement. 2015;1(1):5:
19. Smith J, Dransfield A. Patient and carer involvement in healthcare education, service delivery and research: avoiding tokenism. Royal College of Nursing; 2019.

For peer review only - http://bmjopen.bmj.com/site/about/guidelines.xhtml

18/09/2019
BMJ Open submission

20. Rolfe DE, Ramsden VR, Banner D, Graham ID. Using qualitative Health Research methods to improve patient and public involvement and engagement in research. Research Involvement and Engagement. 2018;4(1):49: 10.1186/s40900-018-0129-8
21. Stableford S, Mettger W. Plain language: a strategic response to the health literacy challenge. Journal of public health policy. 2007;28(1):71-93:
22. Sørensen K, Van den Broucke S, Fullam J, Doyle G, Pelikan J, Slonska Z, et al. Health literacy and public health: A systematic review and integration of definitions and models. BMC Public Health. 2012;12(1):80: 10.1186/1471-2458-12-80
23. Smith SK, Dixon A, Trevena L, Nutbeam D, McCaffery KJ. Exploring patient involvement in healthcare decision making across different education and functional health literacy groups. Social science & medicine. 2009;69(12):1805-12:
24. Nutbeam D. The evolving concept of health literacy. Social science & medicine. 2008;67(12):2072-8:
25. Sørensen K, Pelikan JM, Röthlin F, Ganahl K, Slonska Z, Doyle G, et al. Health literacy in Europe: comparative results of the European health literacy survey (HLS-EU). European Journal of Public Health. 2015;25(6):1053-8:
26. Nutbeam D. Health literacy as a public health goal: a challenge for contemporary health education and communication strategies into the 21st century. Health Promotion International. 2000;15(3):259-67:
27. Batterham R, Hawkins M, Collins P, Buchbinder R, Osborne R. Health literacy: applying current concepts to improve health services and reduce health inequalities. Public Health. 2016;132:3-12:
28. Paasche-Orlow MK, Parker RM, Gazmararian JA, Nielsen-Bohlman LT, Rudd RR. The prevalence of limited health literacy. Journal of general internal medicine. 2005;20(2):175-84:
29. Briant KJ, Halter A, Marchello N, Escareno M, Thompson B. The Power of Digital Storytelling as a Culturally Relevant Health Promotion Tool. Health promotion practice. 2016;17(6):793-801: 10.1177/1524839916658023
30. Skelton SL, Waterman AD, Davis LA, Peipert JD, Fish AF. Applying best practices to designing patient education for patients with end-stage renal disease pursuing kidney transplant. Progress in Transplantation. 2015;25(1):77-90:
31. Christiansen A. Storytelling and professional learning: A phenomenographic study of students' experience of patient digital stories in nurse education. Nurse Education Today. 2011;31(3):289-93:
32. Guyatt GH, Oxman AD, Kunz R, Vist GE, Falck-Ytter Y, Schunemann HJ. What is "quality of evidence" and why is it important to clinicians? Bmj. 2008;336(7651):995-8: 10.1136/bmj.39490.551019.BE
33. Linstone HA, Turoff M. The delphi method: Addison-Wesley Reading, MA; 1975.
34. Sprangers MA, Aaronson NK. The role of health care providers and significant others in evaluating the quality of life of patients with chronic disease: a review. Journal of clinical epidemiology. 1992;45(7):743-60:
35. Sinha IP, Altman DG, Beresford MW, Boers M, Clarke M, Craig J, et al. Standard 5: selection, measurement, and reporting of outcomes in clinical trials in children. Pediatrics. 2012;129(Supplement 3):S146-S52:
36. Dinglas VD, Gifford JM, Husain N, Colantuoni E, Needham DM. Quality of life before intensive care using EQ-5D: patient versus proxy responses. Critical care medicine. 2013;41(1):9-14: 10.1097/CCM.0b013e318265f340
37. Arons AM, Krabbe PF, Schölzel-Dorenbos CJ, van der Wilt GJ, Rikkert MGO. Quality of life in dementia: a study on proxy bias. BMC Medical Research Methodology. 2013;13(1):110: 10.1186/1471-2288-13-110

For peer review only - http://bmjopen.bmj.com/site/about/guidelines.xhtml

Figure 1

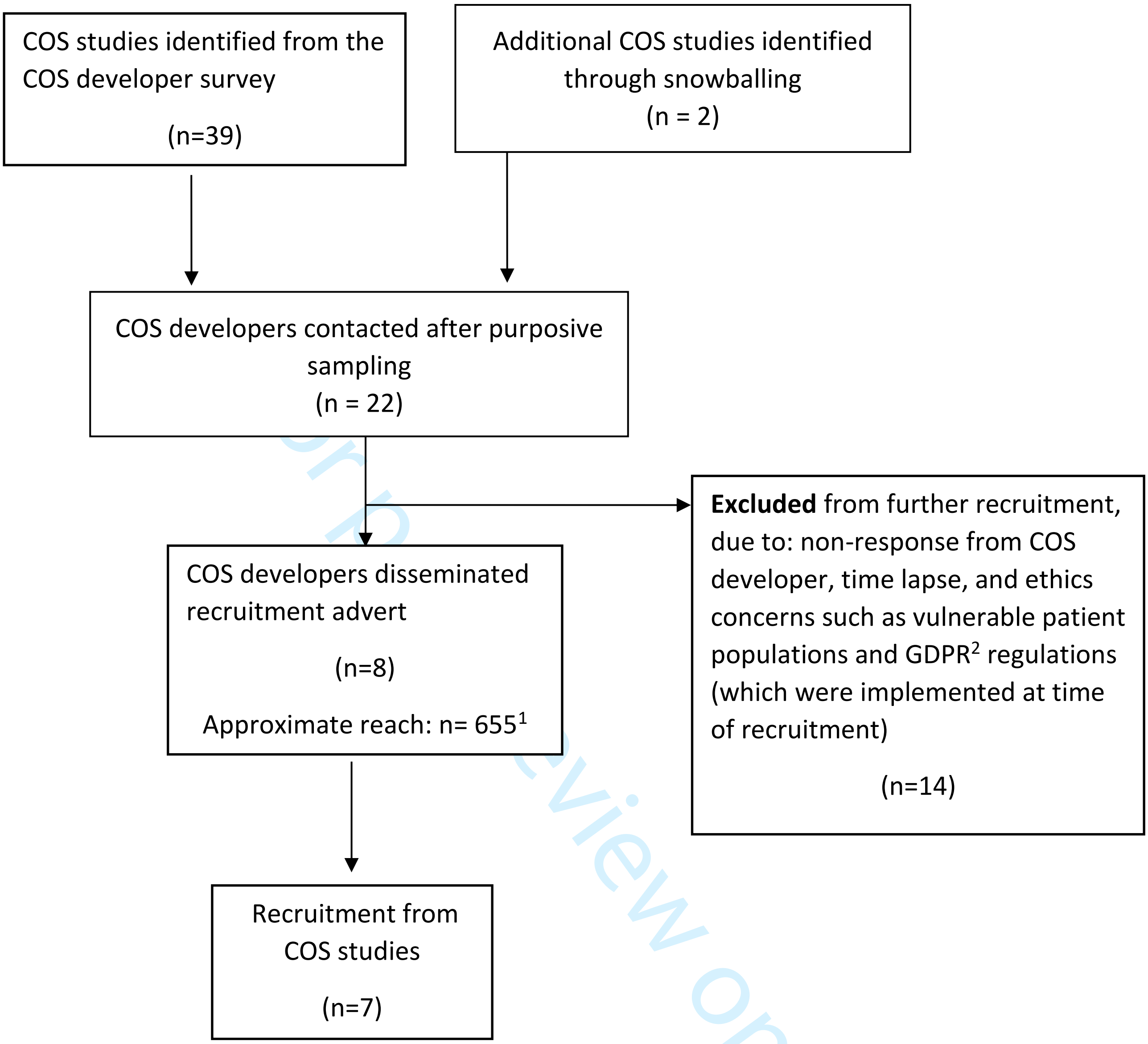

Figure 1: Sampling of COS studies that fit our sampling framework. [1]Reach of two COS studies is unknown, approximate relates to the other 6 COS studies. [2] General Data Protection Regulation (GDPR) is a European Union (EU) law regulation regarding data protection and privacy for all individuals within the EU and the European Economic Area [15]

For peer review only - http://bmjopen.bmj.com/site/about/guidelines.xhtml

Supplementary File 1. Recruitment Advert

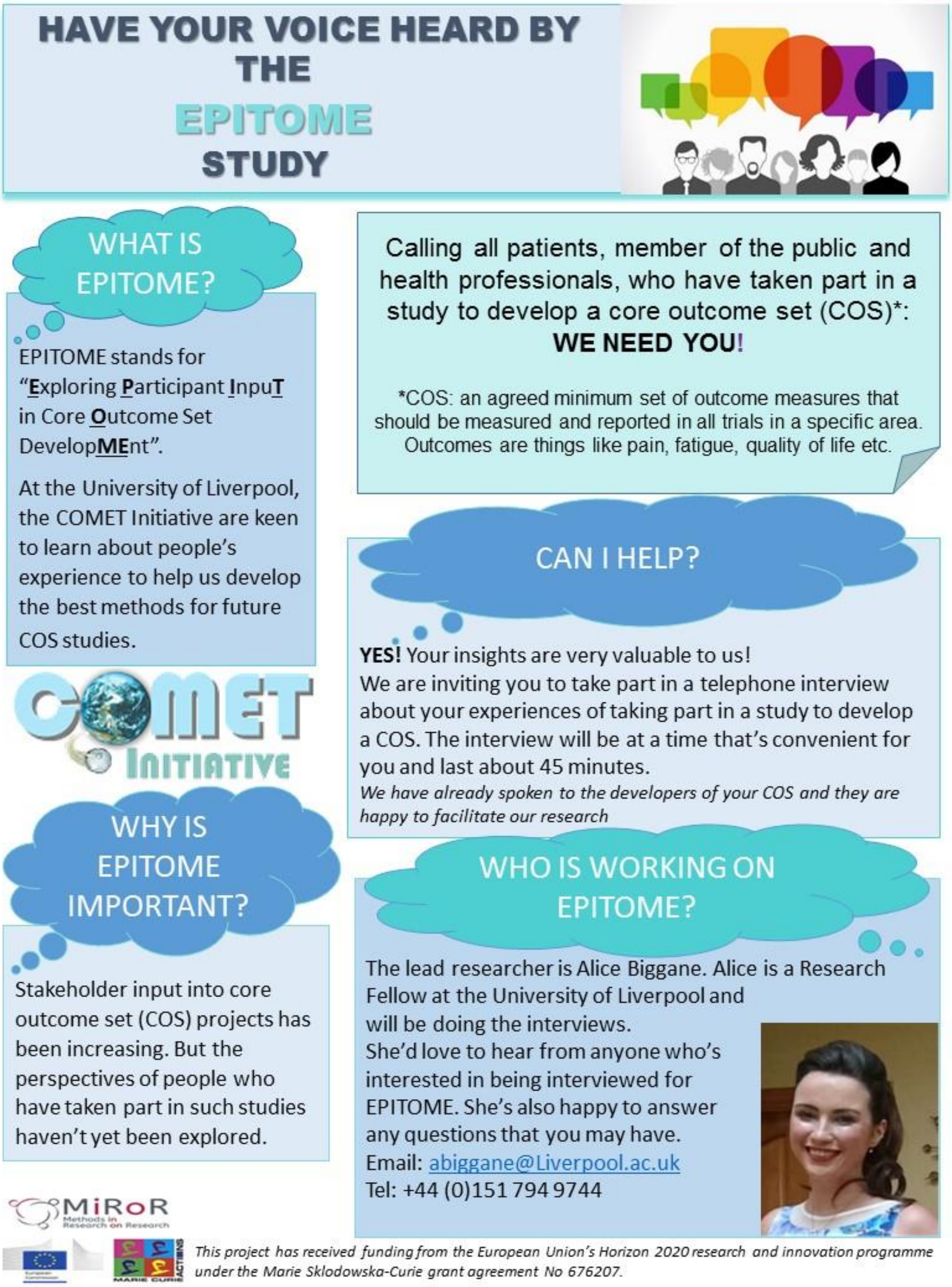

Supplementary File 2. Topic Guide

The idea of this topic guide is that the interviewer will be able to employ cognitive interviewing techniques as much as possible. By asking open and general questions it is hoped that the interviewee will retrospectively recall most of the events without interference from the interviewer, only to clarify certain aspects. However this will not always be the case and as such more detailed questions and prompts are also included. For the "engagement phase" topic of this guide, it should be noted that it may be repeated, depending on how many methods the interviewee was involved in.

Tick list:

Item	Done
Consent Form	
Expected duration of interview	
Introduction/ Explanation of process	

Topic guide (chronological)

Topic	Prompts
Background<br>*Aims: to get interviewee talking and to find out contextual information about how his/her experience of the COS development began.*	• Talk me through how you became involved in the study?<br>• How did you become aware of the study? – *Prompts: recruitment advert, methods*<br>• What were initial thoughts on it? *Prompts: Relevance, worthiness, was it explained adequately etc.*<br>• How did you make the decision to participate?<br>• Feelings about the decision
Preparation<br>*Aims: to understand how the interviewee prepared for the COS development. From their perspective and also how the study developers informed them.*	• Talk me through what happened once you decided to participate? *Prompts: what were the various stages?*<br>• What contact with the COS developers did you have before meeting them? *Prompts: post, phone calls, emails*<br>• Was this contact useful?<br>• Were you supplied with a patient information sheet? Did you look at it? *Prompts: Was it satisfactory? Did you feel like it was explained in terms you could understand?*<br>• How were outcomes described to you? *Prompts: Priorities, effect of research/ effects of treatment on life, lived experience, what is important to the patient/ what matters to them?*<br>• Did you have a clear idea about what was happening? *Prompts: Length of time, process*<br>• Was there support available to you should you need it?

For peer review only - http://bmjopen.bmj.com/site/about/guidelines.xhtml

	• Did you use the support? Was it helpful to you?
Engagement phase<br>*Aims: to elicit information regarding the process itself.*	• Talk me through what happened at the meeting/interview/ focus group/ Delphi etc.?<br>• What methods were used by the developers to elicit your thoughts and perspectives?<br>• What did you think of these methods?<br>○ Were you able to express your thoughts and feelings?<br>○ Do you feel they that your opinions were clearly re<br>• Dis you have any questions about the process? Was there support/someone to help with these? Did you access this support? Did it help?<br>○ In what capacity?<br>• For how long did your involvement in the study run? Or was it a once off?<br>○ Were you comfortable with that length of time?<br>*Prompts: too long, too short, gaps in between contact*<br>• Is there anything you would have liked to change?
Present Day<br>*Aims: to encourage the interviewee to retrospectively analyse their experience; the emotions, the process, whether the process worked or not, suggestions and messages to others.*	• Looking back on the experience what you are your thoughts about it?<br>○ Anything surprised or puzzled you?<br>○ Any suggestions for change-<br>▪ would you do it again<br>▪ would you recommend it to others?<br>• Face to face meetings with health professionals: experiences, concerns, thoughts<br>• Did you receive a copy of the final results *(if the results have been published- interviewer discretion)*?<br>• Messages to others:<br>○ Other participants, academics, developers, health professionals
Other	Anything else that wasn't covered that you think is important?

For peer review only - http://bmjopen.bmj.com/site/about/guidelines.xhtml

Standards for Reporting Qualitative Research (SRQR)*

http://www.equator-network.org/reporting-guidelines/srqr/

Title and abstract

	Page/line no(s).
**Title** - Concise description of the nature and topic of the study Identifying the study as qualitative or indicating the approach (e.g., ethnography, grounded theory) or data collection methods (e.g., interview, focus group) is recommended	Pg. 1/ Line 2-4
**Abstract** - Summary of key elements of the study using the abstract format of the intended publication; typically includes background, purpose, methods, results, and conclusions	Pg.2/ Line 2-25

Introduction

**Problem formulation** - Description and significance of the problem/phenomenon studied; review of relevant theory and empirical work; problem statement	Pg.3/ Line 1-22
**Purpose or research question** - Purpose of the study and specific objectives or questions	Pg.3/ Line 23-30

Methods

**Qualitative approach and research paradigm** - Qualitative approach (e.g., ethnography, grounded theory, case study, phenomenology, narrative research) and guiding theory if appropriate; identifying the research paradigm (e.g., postpositivist, constructivist/ interpretivist) is also recommended; rationale**	Pg.4/ Line 3-5
**Researcher characteristics and reflexivity** - Researchers' characteristics that may influence the research, including personal attributes, qualifications/experience, relationship with participants, assumptions, and/or presuppositions; potential or actual interaction between researchers' characteristics and the research questions, approach, methods, results, and/or transferability	Pg.5/ Line 6-8
**Context** - Setting/site and salient contextual factors; rationale**	Pg.4 / Line 19-20
**Sampling strategy** - How and why research participants, documents, or events were selected; criteria for deciding when no further sampling was necessary (e.g., sampling saturation); rationale**	Pg.4 / Line 7-11<br>Pg. 4/ Line 41<br>Pg.5/ Line 1-4
**Ethical issues pertaining to human subjects** - Documentation of approval by an appropriate ethics review board and participant consent, or explanation for lack thereof; other confidentiality and data security issues	Pg.5/ Line 4-5
**Data collection methods** - Types of data collected; details of data collection procedures including (as appropriate) start and stop dates of data collection and analysis, iterative process, triangulation of sources/methods, and modification of procedures in response to evolving study findings; rationale**	Pg. 4/ Line 17-32

For peer review only - http://bmjopen.bmj.com/site/about/guidelines.xhtml

**Data collection instruments and technologies** - Description of instruments (e.g., interview guides, questionnaires) and devices (e.g., audio recorders) used for data collection; if/how the instrument(s) changed over the course of the study	Pg. 4 Line 17-32
**Units of study** - Number and relevant characteristics of participants, documents, or events included in the study; level of participation (could be reported in results)	Pg.6 /Line 1-15<br>Pg.7 / Line 25-33<br>Pg. 8/ Line 1-6
**Data processing** - Methods for processing data prior to and during analysis, including transcription, data entry, data management and security, verification of data integrity, data coding, and anonymization/de-identification of excerpts	Pg. 5/ Line 31-32
**Data analysis** - Process by which inferences, themes, etc., were identified and developed, including the researchers involved in data analysis; usually references a specific paradigm or approach; rationale**	Pg. 4/ Line 34-39
**Techniques to enhance trustworthiness** - Techniques to enhance trustworthiness and credibility of data analysis (e.g., member checking, audit trail, triangulation); rationale**	Pg. 4/ Line 34-41

Results/findings

**Synthesis and interpretation** - Main findings (e.g., interpretations, inferences, and themes); might include development of a theory or model, or integration with prior research or theory	Pg. 12-16 inclusive
**Links to empirical data** - Evidence (e.g., quotes, field notes, text excerpts, photographs) to substantiate analytic findings	Pg.12 -16 inclusive

Discussion

**Integration with prior work, implications, transferability, and contribution(s) to the field -** Short summary of main findings; explanation of how findings and conclusions connect to, support, elaborate on, or challenge conclusions of earlier scholarship; discussion of scope of application/generalizability; identification of unique contribution(s) to scholarship in a discipline or field	Pg.18/ Line 1-42<br>Pg.19 / Line 1-42<br>Pg. 20/ Line 1-16
**Limitations** - Trustworthiness and limitations of findings	Pg.20 /Line 22-27

Other

**Conflicts of interest** - Potential sources of influence or perceived influence on study conduct and conclusions; how these were managed	Pg. 21/ Line 14-16
**Funding** - Sources of funding and other support; role of funders in data collection, interpretation, and reporting	Pg. 21 /Line 21-24

*The authors created the SRQR by searching the literature to identify guidelines, reporting standards, and critical appraisal criteria for qualitative research; reviewing the reference lists of retrieved sources; and contacting experts to gain feedback. The SRQR aims to improve the transparency of all aspects of qualitative research by providing clear standards for reporting qualitative research.

For peer review only

For peer review only - http://bmjopen.bmj.com/site/about/guidelines.xhtml

**The rationale should briefly discuss the justification for choosing that theory, approach, method, or technique rather than other options available, the assumptions and limitations implicit in those choices, and how those choices influence study conclusions and transferability. As appropriate, the rationale for several items might be discussed together.

Reference:

O'Brien BC, Harris IB, Beckman TJ, Reed DA, Cook DA. **Standards for reporting qualitative research: a synthesis of recommendations.** *Academic Medicine*, Vol. 89, No. 9 / Sept 2014 DOI: 10.1097/ACM.0000000000000388

For peer review only
